# Nanoquinacrine induced apoptosis in cervical cancer stem cells through the inhibition of hedgehog-GLI1 cascade: Role of GLI-1

**DOI:** 10.1038/srep20600

**Published:** 2016-02-05

**Authors:** Anmada Nayak, Shakti Ranjan Satapathy, Dipon Das, Sumit Siddharth, Neha Tripathi, Prasad V. Bharatam, ChanakyaNath Kundu

**Affiliations:** 1 Cancer Biology Division, KIIT School of Biotechnology, KIIT University, Campus-11, Patia, Bhubaneswar, Orissa, 751024, India; 2 Department of Pharmacoinformatics, National Institute of Pharmaceutical Education and Research (NIPER), Sector 67, S.A.S. Nagar, Mohali, Punjab 160062, India; 3 Department of Medicinal Chemistry, National Institute of Pharmaceutical Education and Research (NIPER), Sector 67, S.A.S. Nagar, Mohali, Punjab 160062, India

## Abstract

To improve the pharmacokinetics and to study the anti-cervical cancer and anti-stem cells (CSCs) mechanism of Quinacrine (QC), a spherical nano particle of QC (i.e. NQC) was prepared and characterized. QC and NQC showed higher cytotoxicity in multiple cancer cells than the normal epithelial cells. NQC exhibited more toxicity in cervical cancer cells and its CSCs than QC. A dose-dependent decreased expression of Hedgehog-GLI (HH-GLI) components were noted in NQC treated HeLa cells and its CSCs. NQC increased the expressions of negatively regulated HH-GLI components (GSK3β, PTEN) and caused apoptosis in CSCs. Reduction of GLI1 at mRNA and promoter level were noted after NQC exposure. The expressions of HH-GLI components, GLI1 promoter activity and apoptosis were unaltered in NQC treated GLI1-knockdown cells. *In silico*, cell based and *in vitro* reconstitution assay revealed that NQC inhibit HH-GLI cascade by binding to the consensus sequence (5′GACCACCCA3′) of GLI1 in GLI-DNA complex through destabilizing DNA-GLI1 complex. NQC reduced the tumors size and proliferation marker Ki-67 in an *in vivo xenograft* mice model. Thus, NQC induced apoptosis in cancers through inhibition of HH-GLI cascade by GLI1. Detail interaction of QC-DNA-GLI complex can pave path for anticancer drug design.

Cervical cancer is a very common form of cancer and a leading cause of subsequent death occurring in women^1^,^2^. Persistent infection, due to Human Papilloma virus (HPV16, HPV18), is one of the important etiological factors for cervical cancer^3^. The discovery of the Gardasil and Ceravix vaccines provided an opportunity in combating cervical cancer in developed nations. However, due to high cost, low cervical cancer screening rate, failure of early detection and lack of impact on existing infection, vaccination remained the major challenge in low income and developing countries^4^. Despite the use of surgery, chemotherapy and vaccines, recurrence rate of cervical cancer is around 35% which indicate that some cells especially the cancer stem cells (CSCs) in the tumor microenvironment sequester themselves from the therapy by some mechanism, probably related to drug absorption or efflux. Due to high drug efflux, DNA repair and self-renewable capabilities, CSCs often escape from the toxic effect of drug. CSC research is a necessary step towards the understanding of tumor growth, recurrence, metastasis and chemo-resistance^5^.

A number of natural, semi-synthetic or synthetic agents are employed for the anticancer therapy. One such interesting example is quinacrine (QC) which possesses a number of desirable properties. QC (a 9-aminoacridine derivative), a synthetic substitute of quinine (obtained from bark of cinchona tree), exhibits anticancer activity against a wide range of cancers including lung, colon, pancreatic, cervical and renal cell carcinoma^6^,^7^. QC is a popular DNA damaging agent, possessing its action through DNA intercalation^8^. Earlier, we have reported anticancer potential of QC through (i) inhibition of topoisomerase activity^9^, (ii) the activation of tumor suppressor gene p53^10^, (iii) the activation of cyclin-dependent kinase inhibitor p21^10^, (iv) induction of autophagy^10^,and (v) inhibition of WNT/TCF signalling^11^. Number of studies indicates that QC exhibits therapeutic potential against malaria, amoebiasis, parasitic infections, giardiasis, systemic lupus erythematosus and prion diseases^12^,^13^,^14^,^15^,^16^,^17^. Interestingly, it was shown that QC inhibits replication of multiple viral genes such as hepatitis C (HCV), encephalomyocarditis (EMCV), polio, tomato bushy stunt (TBSV) and HIV^18^. It has also been reported that QC dose-dependently inhibits EMCV and polio virus-infected HeLa cells, viral capsid protein synthesis, production of viral RNAs, and virus replication^18^. Unfortunately, regardless of all the advantages, QC suffers from clinical limitations like yellow pigmentation on skin and poor bioavailability^13^. Liposome encapsulated QC has been reported to cause apoptosis in breast and glioma CSCs^19^,^20^. These studies point out that poor absorption of quinacrine in CSCs can be overcome by designing appropriate drug delivery systems. Nano-formulation can probably, help to improve the pharmacokinetic profile of QC, especially the low water solubility and poor bioavailability.

QC acts as a cytotoxic agent through a number of mechanisms which are not yet completely explored. This demands identification of the target signalling pathway, as its inhibition leads to cellular apoptosis; which further requires better understanding of various constituents of cancer. Invasion and metastasis are two important features for cancer progression. Metastasis is a complex process of sequential events characterized by tumor cell detachment, Epithelial to mesenchymal transition (EMT), intravasation, survival within blood and lymphatic vessel, mesenchymal to epithelial transition, micrometastasis and finally macrometastasis^21^. Reports suggest hypoxia stimulated downregulation of E-cadherin (maintains the epithelial polarity) is a functional requirement for epithelial to mesenchymal transition^22^. Furthermore, downregulation of E-cadherin is considered as an important step during metastasis and cancer progression^23^. Further, various signaling macromolecules e.g. WNT, Hedgehog-GLI (HH-GLI) and NOTCH are involved in cancer metastasis. In normal adult physiology, HH-GLI signaling pathway is implicated in stem cell maintenance, tissue repair and regeneration. The deregulation of HH-GLI pathway leads to diverse spectrum of cancers^24^,^25^,^26^. HH-GLI signaling pathway begins with the interaction of post translational modified HH ligand Sonic Hedgehog (SHH) with Patched-1 (PTCH1) receptor. In the absence of HH-GLI signalling, PTCH1 binds to a seven trans-membrane pass protein Smoothened (SMO) to inhibit its signaling function^27^. SHH interaction with PTCH1 relieves the inhibition of SMO which in turn leads to the activation of GLI group of transcription factors. Activator GLI (GLI1 and GLI2) and repressor GLI (GLI3) translocate to the nucleus and modulate the transcription of HH target genes e.g. cyclin D1, cMyc, etc^28^. Although, GLI group of transcription factors are the major modulators of HH-GLI pathway, it is also known to be activated non-canonically by other signaling molecules e.g. RAS, RAF, MEK, ERK, etc. Synthetic agents (e.g. GDC-0449, IPI-926, GANT61) and siRNA based GLI knockdown leads to tumor disappearance, inhibition of tumor recurrence both *in vitro* and *in vivo*^29^. GANT61, a specific inhibitor of GLI1 reduces cell viability, spheroid formation, GLI-DNA binding, transcriptional activities and induces apoptosis by caspase 3 activation, PARP cleavage. It also up-regulated the death receptors TRAIL-R1/DR4, TRAIL-R2/DR5 and Fas in pancreatic cancer stem cells^30^,^31^. GLI1 is reported to interact with DNA via a consensus promoter sequence (5′GACCACCCA3′) which in turn induces the transcription of target genes^32^. Previously, using surface plasmon resonance and molecular docking, Agyeman *et al*. have shown that GANT61 binds to GLI1 near Glu119 and Glu167, between Zinc fingers domain 2 and 3 instead of conserved promoter sequence or GLI-DNA complex^33^.

In the present work, application of nano-formulation strategy to improve the bioavailability of QC at the site of action is demonstrated. Further, the anti-cancer potential of QC (using NQC) against cervical cancer cells and CSCs is evaluated specifically in terms of its mechanism of action. The cell based assays, *in vivo xenograft* along with the molecular modelling studies were used to identify the target signalling pathway for the anticancer and cytotoxic activity of QC. *In silico* studies were specifically employed to understand the atomistic details of the QC interaction with components of HH-GLI pathway.

## Results

### Synthesis and Characterization of NQC

**Figure 1:**
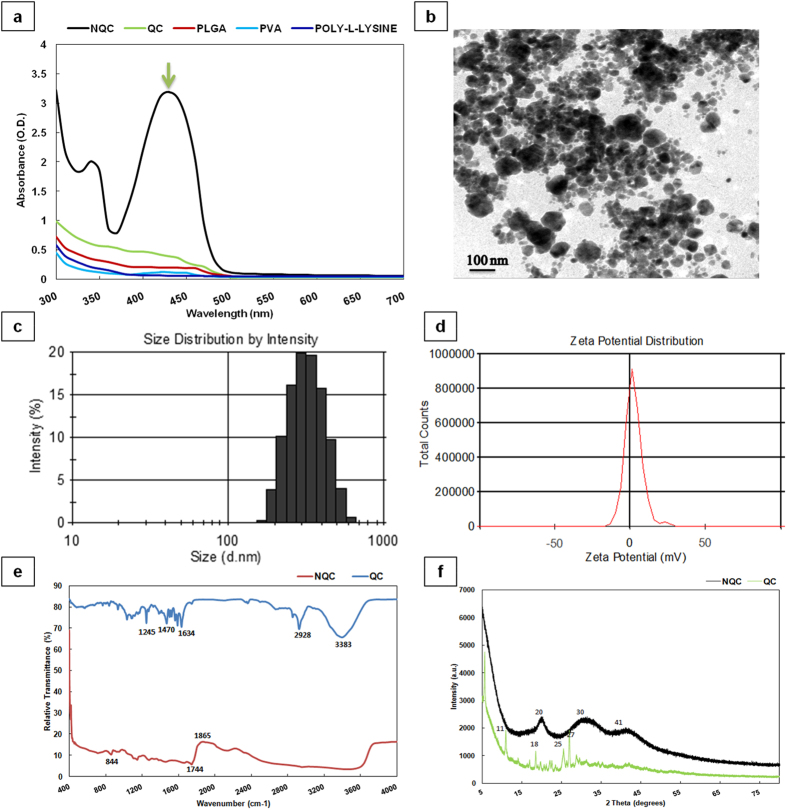
Characterization of NQC. (**a**) UV-Visible spectrum of NQC, QC, PLGA, PVA and Poly-L-Lysine. (**b**) Transmission electron micrograph (TEM) of NQC. (**c**) Size distribution analysis of NQC. (**d**) Zeta potential analysis showing surface charge distribution of NQC. (**e**) FT-IR spectra of NQC and QC. (**f**) XRD pattern of NQC and QC.

To improve the bioavailability, pharmacokinetics as well as *in vivo* therapeutic efficiency, a Poly-lactic-co-glycolic acid (PLGA) encapsulated nano form of QC (i.e. NQC) was synthesized and characterized for morphology, size, and surface charge distribution (Fig. 1). Fig. 1a shows the UV-Vis absorption spectrum of different agents. The maximum absorption at 420 nm with a characteristic peak was exhibited by NQC indicated a stable, homogeneous nano-formulation with narrow size particle distribution. There was no sign of precipitation during storage for more than 4–6 months. Approximately 100 nm diameter of particle size was noted by TEM (Fig. 1b).

The size of NQC showed a mean diameter of 291.400 ± 0.040 d.nm with a polydispersity index (PDI) of 0.127 ± 0.020 (Fig. 1c). Zeta potential was measured to know the surface charge distribution of the particle. NQC has a zeta potential of +2.380 ± 0.920 mV (Fig. 1d). FT-IR analysis of nanoparticles with the polymer matrix showed significant peaks at 1865, 1744 cm^−1^ while the spectra for the QC showed peaks at 1245, 2928 and 3383 cm^−1^ (Fig. 1e). The characteristic peak of QC at 3383 cm^−1^ completely disappeared in NQC indicating a change in N-H stretch. Further, the peak at 1634 cm^−1^ in QC spectra was shifted to 1744 cm^−1^ in NQC pointing towards an N-H bend and change in amine group. To study the effect of PLGA entrapment on the crystalline behaviour of NQC, XRD analysis was performed and compared with that of QC and was found that NQC is nearly face centred cubic (FCC) in compared to parental counterpart (Fig. 1f).

### NQC caused apoptosis in cervical cancer cells

To measure the cytotoxic potential of NQC in different cancer cell lines, including well established three cervical cancer cell lines and two normal cell lines, the cell proliferative activity was measured using MTT assay and data was compared with QC. Cells were exposed to various concentrations of QC and NQC for different time intervals and assay was performed. Although both QC and NQC decreased cell proliferation with time, optimal cell death was noticed at 48 h. Beyond 48 h, no significant increase in cell death was noticed. Thus, 48 h was considered as optimal time for QC/NQC exposure for cell based assays.

**Figure 2:**
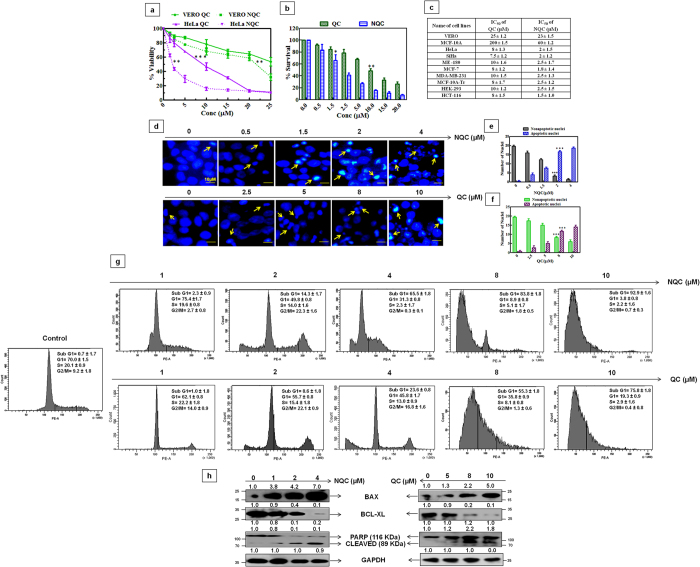
NQC is more cytotoxic in cervical cancer cells in comparison to normal epithelial cells. Cells were treated with different concentrations of QC and NQC and assays were carried out according to materials and methods described in the text. (**a**) MTT cell viability assay in HeLa and Vero cells. (**b**) Clonogenic cell survival assay in HeLa cells. (**c**) Tabular representation of IC_50_ values of QC and NQC in different cells. (**d**) Detection of apoptosis by DAPI nuclear staining. Images (scale bar 10 μm) were taken with fluorescence microscope with 40× magnification (Nikon, Japan). Graphical representation of apoptotic and non-apoptotic DAPI stained nuclei in NQC (**e**), and QC (**f**) treated HeLa cells. (**g**) Regulation of cell cycle profile in HeLa cells. (**h**) Representative expressions of pro- and anti-apoptotic markers in HeLa whole-cell lysate. GAPDH severed as loading control. Data from quantification immunoreactive signal is represented as a fold to control and statistical significance was determined by paired *t* test, (*p < *0.05*), (**p < 0.005), (***p < 0.001).

The dose-dependent cell viability was analyzed using same MTT assay. Figure 2a demonstrates the cell viability profile of QC and NQC in cervical cancer cells HeLa and normal epithelial cells Vero. It was observed that QC and NQC exhibited higher cytotoxicity in HeLa cells as compared to Vero cells. The IC_50_ (concentration at which 50% growth of cells inhibit in culture) in HeLa cells was 8 μM and 2 μM for QC and NQC, respectively. In Vero cells, similar effect (IC_50_) was observed at 25 μM QC and 23  M NQC, respectively.

Cancer cells exhibit the hallmark property of indefinite proliferation i.e. a single cell retains its reproductive ability and thus multiply indefinitely to form a colony or clone. The inhibitory effect of QC and NQC on colony formation ability in HeLa cells was assessed by clonogenic assay. A dose-dependent decrease in colony formation was noticed in QC as well as NQC treated cells. The LC_50_ (concentration at which 50% cell death occur in culture) was noted at 9 μM and 1.8 μM with QC and NQC, respectively (Fig. 2b). It was noticed that NQC caused more cytotoxicity in all the chosen cancer cells types (e.g. breast (MCF7, MDA-MB-231, MCF-10A-Tr), colon (HCT-116), kidney (HEK-293) and cervical (SiHa, ME-180) in comparison to normal epithelial cells, MCF-10A (breast) and Vero (kidney) (Fig. 2c).

To identify the cause behind inhibition of cellular proliferation, the nuclei of QC and NQC treated cells were observed after fixing with DAPI. A dose-dependent increase in shrunken, bean shaped and fragmented nuclei were observed in QC and NQC treated cells (Fig. 2d). Graphical representation in Fig. 2e,2f showed a comparison of apoptotic and normal nuclei in NQC and QC treated cells, respectively. Thus, NQC caused cell death at lower concentrations than QC, which is in accordance with IC_50 _data obtained. To confirm DAPI nuclear staining result, FACS analysis was performed using Propidium iodide (PI) staining. An increase in the population of Sub-G_1_ was observed with increasing NQC treatment. Interestingly, 75.8% and 92.9% apoptosis was seen in 10 μM QC and 10 μM NQC treated cells, respectively. Interestingly, a significant G_2_/M arrest was noted in NQC treated cells but not in QC treated cells (Fig. 2g).

To further confirm apoptosis, the expressions of multiple pro- and anti-apoptotic protein markers were analyzed in QC and NQC treated cellular lysates. Increased expressions of BAX/BCL-xL ratio, cleaved PARP were noted with increased dose of QC and NQC with a higher efficacy of NQC than QC (Fig. 2h). From the above data, it is evident that both QC and NQC caused cervical cancer cell death and NQC have a higher apoptotic potential than QC.

### NQC targets HH-GLI axis for apoptosis

**Figure 3:**
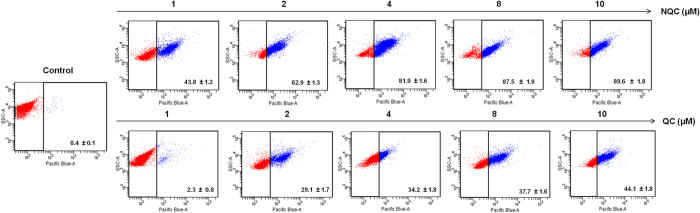
Cellular uptake of QC and NQC in HeLa cells. Data presented here is mean ± SD of three independent experiments and statistical significance was determined by paired’t’ test, (***p < 0.001).

**Figure 4:**
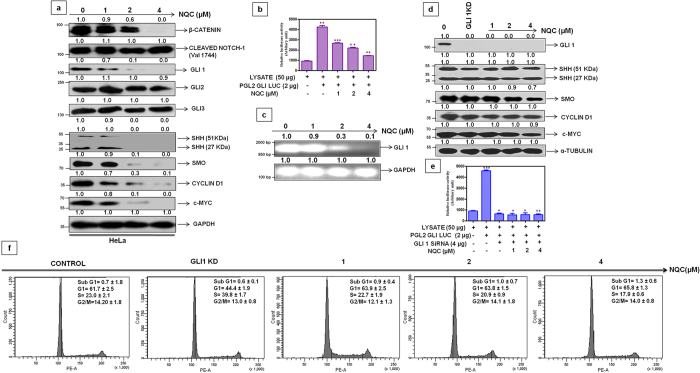
Inhibition of HH-GLI cascade by NQC in HeLa cells is through GLI1. Cells were treated with different concentrations of NQC for 48 h and then assays were carried out according to materials and methods described in text. (**a**) Expressions of representative proteins of WNT-TCF, HH-GLI and NOTCH signaling cascade in the whole cell extracts. GAPDH serves as loading control. (**b**) GLI1 promoter activity. Luciferase activity was measured after transiently transfecting PGL2-GLI-luc promoter plasmid followed by NQC treatment (**c**) GLI-mRNA expressions measured by quantitative RT PCR. GAPDH serves as loading control. (**d**) Expressions of proteins of HH-GLI signaling after NQC treatment in HeLa GLI1 knock down cells. α - tubulin serves as loading control. (**e**) GLI1 promoter activity in HeLa GLI1 knock down cells. GLI1 knock down cells were transiently transfected with PGL2-GLI1-luc promoter plasmid and then luciferase activity was measured after NQC exposure. (**f**) Regulation of cell cycle profile in NQC treated HeLa GLI1 knock down cells. Data is the representation of three independent experiments. Statistical significance was determined by paired *t* test (*p < 0.05), (**p < 0.005), (***p < 0.001).

To decipher the mechanism of action for the apoptosis caused by NQC, several cell based assays were employed. First, we have analyzed the cellular uptake of QC and NQC in HeLa cells using FACS. A dose dependent increased accumulation of NQC into the cells was noted but no significant increased of QC accumulation was observed. The uptake of NQC is very high (MFI = 43.8%) even at low dose of exposure (1 μM) while QC showed an uptake of 2.3% at the same dose (Fig. 3). At 10 μM NQC and QC maximum of 89.6% and 44.1% MFI, respectively, was observed (Fig. 3). Then, we wanted to check whether NQC targets one of the major signalling cascades (e.g. HH-GLI, WNT-TCF and NOTCH) for metastasis. β-catenin, transcriptional activator of WNT-TCF signaling, decreased only at a very high concentration of NQC, whereas no change in the expression of cleaved NOTCH 1 (Val1744), a transcriptional activator of NOTCH cascade was noted (Fig. 4a). Interestingly, it was found that GLI1 (key intermediate of HH-GLI cascade) decreased in a dose-dependent manner, without appreciable changes in the expressions of other GLI isoforms (i.e. GLI 2, GLI 3) (Fig. 4a).

To evaluate the impact of NQC on HH-GLI pathway, the protein expressions of intermediates in HH-GLI signaling were measured in two cervical cancer cell lines e.g. HeLa (Fig. 4a) and SiHa (Supplementary Fig. S1). A dose-dependent decrease in the expressions of SHH, SMO, GLI1, c-MYC and CYCLIN D1 in both the cell lines were noted (Fig. 4a and supplementary Fig. S1). To check the effect of NQC in GLI1 transcription factor, we measured the GLI1 promoter activity and mRNA expression after NQC treatment in HeLa cells. A dose-dependent decrease of luciferase activity (PGL2-GlI-Luc) was observed in NQC treated cells. Relative luciferase activity reduced to 50% at 2 μM NQC compared to control (Fig. 4b). Semi-quantitative PCR in NQC treated HeLa cell also revealed a dose-dependent decrease (7 fold in compare to untreated cells) in mRNA expressions of GLI1 (Fig. 4c).

To confirm the down-regulation of GLI1 as the cause of NQC mediated cell death, the GLI1 was silenced in HeLa cells and experiments were carried out. The GLI1 expression was completely abolished in GLI1 knockdown (GLI1-KD) cells. The protein expressions of SHH, SMO, CYCLIN-D1, c-MYC and GLI1 promoter activity were unaltered in NQC treated GLI1-KD cells (Figs 4d,4e). Interestingly, no significant change in Sub G_1_ was noted in NQC treated GLI1-KD cells after FACS analysis (Fig. 4f). Thus, data appears that NQC mediated cell death in HeLa cells is HH-GLI dependent.

### NQC reduces cell proliferation in cervical CSCs

**Figure 5:**
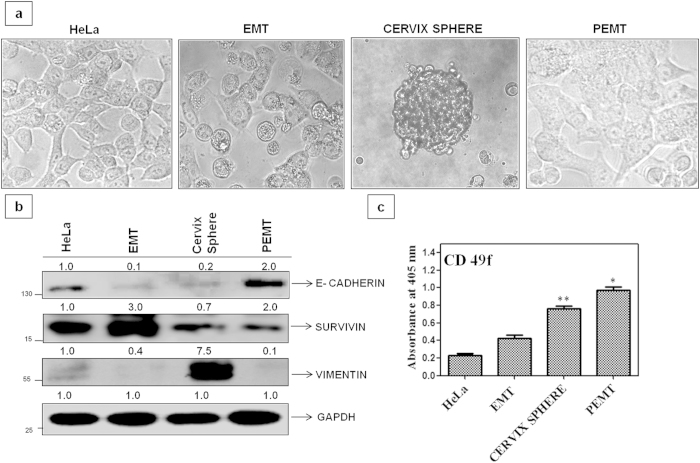
Development and characterization of cervical-CSC model in HeLa cells. (**a**) Morphology of different metastatic phases of cervical cancer cells. Photograph was taken in a bright field microscope at 20× magnification. (**b**) Expressions of stemness markers in different metastatic phases. GAPDH serves as loading control. (**c**) Expressions of cervical-CSC marker CD 49f in different phases of metastasis. Data presented here is mean ± SD of three independent experiments. Statistical significance was determined by paired *t* test (*p < 0.05), (**p < 0.005).

Cancer stem cells (CSC) are now ranked in the top of hierarchy proved to be appealing towards disease recurrence, chemo and radiation resistance. To study the efficacy of NQC against CSCs, a model system was developed using HeLa cells according to protocol described in methods and material (Fig. 5a). Epithelial to mesenchymal transition (EMT) and its reverse phenomena (Mesenchymal to epithelial transition; MET) is the hallmark of metastasis. Culture of cells in serum deprivation/hypoxic medium for 6 to 8 days led to the development of a pre-metastatic phase known as EMT with morphology different from the parental cell (Fig. 5a). Culture in the same medium led to the development of visible sphere like viable cluster known as spheroids (cervix sphere). When these spheroids were given back to the serum affluent condition, they grew as adherent monolayer and grew more aggressively than parental cells are known as PEMT which have already undergone epithelial to mesenchymal transition (Fig. 5a). Loss of E-Cadherin (an epithelial marker), elevated Vimentin (a mesenchymal marker) in the cervix sphere (MET) and increased expression of Survivinin EMT (Fig. 5b) as well as increased CD-49f during transition of EMT to PMET confirmed the metastasis model (Fig. 5c).

**Figure 6:**
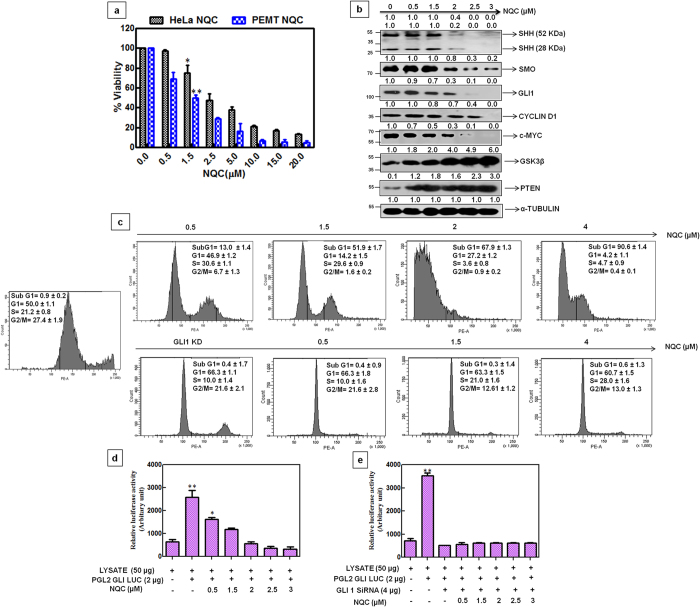
NQC caused cytotoxicity in cervical CSCs. Cells were exposed to different concentrations of NQC for 48 h and then different assays were carried out. (**a**) Comparative cell survival of PEMT and HeLa cells. (**b**) Change in expression of HH-GLI components at protein level in PEMT cells. GAPDH was used as the loading control. (**c**) Cell cycle profile of NQC exposed PEMT cells. (**d**,**e**) GLI1 reporter activity in PEMT and GLI1-knock down PEMT cells after NQC treatment, respectively. Data presented is the mean ± SD of three independent experiments. Statistical significance was determined by paired *t* test (*p < 0.05), (**p <* *0.005).

The anti-proliferation effect of NQC was measured in PEMT and compared with parental HeLa cells using MTT assay. IC_50_ was noted at 1.5 μM and 2.5 μM in PEMT and HeLa cells, respectively after exposure to NQC (Fig. 6a). The effect of NQC on major signaling cascade of CSCs (HH-GLI signaling) was studied by examining the expressions of various proteins involved in this signaling in PEMT cells. A dose-dependent decrease of SHH, SMO, GLI1 were noted in NQC exposed PMET cells (Fig. 6b). Interestingly, the negative regulator of HH-GLI cascade, GSK3β and tumour suppressor protein, PTEN dose dependently increased after NQC treatment. A reduction in the expressions of c-MYC and Cyclin-D1, downstream target GLI1 were also noted (Fig. 6b). The regulation of cell cycle and apoptotic potential of NQC in PEMT cells were measured by FACS analysis. The percentage of cells in S and G_1_ phase decreased while cells at sub-G_1_ phase increased in a dose-dependent manner with NQC exposure. More than 90% cells were in sub-G_1_ phase at 4 μM NQC treatment. But no significant apoptosis was observed after NQC treatment in GLI-KD-PEMT cells (Fig. 6c). To conclude whether the NQC mediated cell death in PEMT cells was through inhibition of GLI1 or not, a luciferase activity of GLI1was measured after over expressing GLI1 reporter plasmid (PGL2- GLI- Luc) followed by treatment with NQC. A dose-dependent reduction of reporter activity was noted after NQC exposure (Fig. 6d). The un-alteration of GLI1 promoter activity in GLI1-silenced NQC-treated PEMT cells with respect to un-transfected control confirmed the GLI1 dependency of NQC mediated apoptosis (Fig. 6e).

**Figure 7:**
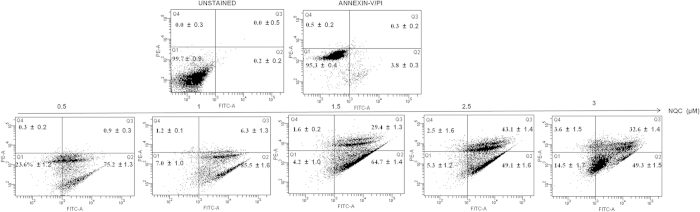
NQC caused apoptosis in PEMT cells. Annexin-V-FITC/PI dual staining in NQC treated PEMT cells. Data presented here is the representative of three independent experiments. Statistical significance was determined by paired *t* test (*p < 0.05).

To confirm that NQC mediated cell death was due to necrosis or apoptosis; Annexin-V-FITC and PI dual staining method was performed using FACS. According to the principle, PI binds to apoptotic and necrotic cells but Annexin binds to the living cells. Fig. 7 demonstrated the distribution of cells in different quadrants. Q1, Q2, Q3 and Q4 represent the live cells, early apoptotic, late apoptotic/necrotic and necrotic cells, respectively. A dose dependent increase of late apoptotic/necrotic cell (Q3) was noted with an increase in NQC exposure. More than 43% late apoptotic/necrotic (Q3) were noticed with a significant increased in necrotic population (Q4; 2.5% with respect to control) at 2.5 μM NQC treated (Fig. 7). The Caspase 3 expression in NQC treated cells was measured by immunofluorescence and was observed to be increasing dose-dependently after NQC treatment (Supplementary Fig. S2). These observations confirmed that the anti-cancer potential of NQC against CSCs is also mediated through GLI1.

### QC inhibits the HH-GLI by binding to GLI in GLI-DNA complex

**Figure 8:**
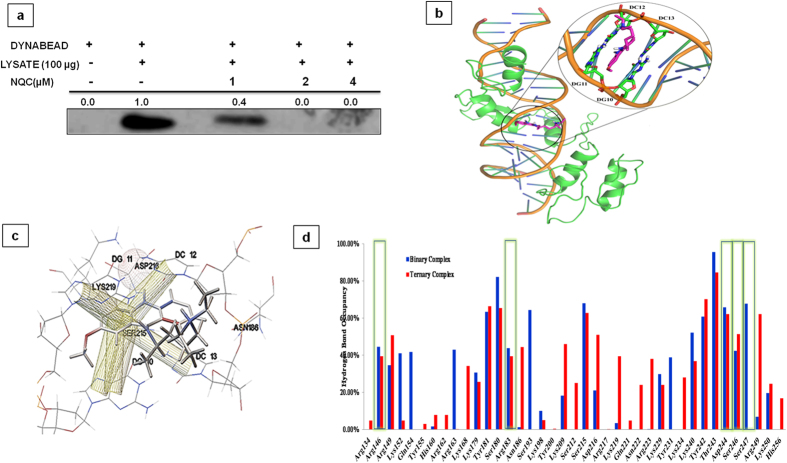
DNA-GLI-QC ternary complex after equilibration run of 1 ns. (**a**) Dynabead mediated GLI-DNA complex pull down assay after treatment with NQC. (**b**) Acridine ring of QC is inserted between the GC base pairs. (**c**) Interaction diagram of QC in DNA-GLI complex. Yellow cylindrical contacts represent π-π interactions and spheres represent the hydrogen bonding interaction (**d**) The hydrogen bond (between DNA and Protein) occupancy analysis for the binary (DNA-GLI, blue) and ternary (DNA-GLI-QC, red) complexes. The residues in boxes are forming hydrogen bonds in the crystal structure (PDB ID: 2GLI).

GANT61 has been shown to inhibit HH-GLI by interacting with GLI1 through the zinc finger 2 and zing finger 3 motifs, without interacting with DNA^33^. The anticancer profile of QC is similar to that of GANT61 and hence it may be hypothesized that QC interact with the DNA-GLI complex in a similar manner to that of GANT61. Based on these observations, a number of possible mechanisms of action for QC have been proposed such as (i) QC may interact with GLI1 and thus leading to the inhibition of its biological function and/or (ii) it can bind to the DNA-GLI1 complex and thus destabilize the same leading to proteasomal degradation. To provide a possible explanation to the effect of QC, *in vitro, in silico* and cell based assays were carried out. In order to determine the fate of GLI1 after NQC treatment, the cells were pre-treated with MG-132 (a proteosomal degradation inhibitor) prior to NQC treatment. Restoration of the GLI1 level suggested that GLI1 moves towards proteosomal degradation after destabilized from DNA-GLI complex (supplementary Fig. S3a). An *in vitro* assay, using two plasmid DNA, one with GLI1 binding consensus sequence (PGL2-GLI1 promoter) (supplementary Fig. S3b) and other without it (WT APC cDNA) was carried out to check the efficacy of binding of NQC to the consensus GLI1 binding sequence. Different concentrations of NQC were incubated with fixed concentration of DNA and absorbance was recorded at different wavelengths. According to the principle the DNA interaction to NQC will lead to hypochromic shift *i.e.* decrease in absorbance and shift to the left. Interestingly, it was noted that NQC strongly binds to PGL2-GLI1 promoter with *K*_*d*_ value 2.5 × 10^−6^ M^−1^ (supplementary Fig. S3c and supplementary Fig. S3d) but does not bind to WT APC (supplementary Fig. S3e). Further, a dynabead mediated GLI-DNA pull down assay was carried out after treating the cells with NQC. GLI1 after translocation into nucleus binds to the consensus 9 nucleotide sequences i.e. 5′GACCACCCA3′. The GLI-DNA complex was pulled down and the expression of GLI1 was measured by western blot. A decrease in expression of GLI1 confirmed that QC inhibits the function of GLI1 by directly binding to the GLI1 consensus sequence in the DNA (Fig. 8a).

In order to evaluate how QC destabilizes the DNA-GLI1 complex, molecular docking studies were carried out using the crystal structure of DNA-GLI binary complex (PDB ID 2GLI) as macromolecular structure and QC (PubChem ID-CID237) as the small molecule. The structures and electrostatic potentials of the DNA and GLI1 are not independently complementary to QC. However, the structure of the cavity formed due to DNA-GLI1 complex formation and the corresponding electrostatic potential are complementary to QC. Hence, the possibility of QC binding to DNA-GLI1 complex can be envisaged (supplementary Fig. S4 and supplementary Fig. S5). To verify this hypothesis, molecular docking of QC in the DNA-GLI complex has been carried out (glide docking score: -6.431) (supplementary Fig. S6). The pose obtained was not very comfortable within the available space (highly strained structure due to bumping and unwanted interactions, especially no intercalation was noticed as expected). This problem arose because the available structure doesn’t provide enough space for intercalation (Supplementary Fig. S7a) which prompted us to make efforts to reposition the QC so as to create necessary space for intercalation.

Manual repositioning of QC between the GC base pairs (supplementary Fig. 7b) led to many close contacts between the GC base pairs and QC which were removed by molecular dynamics simulation. Figure 8b shows the equilibrated DNA-GLI-QC ternary complex. The QC shows π-π interactions with the dG10···dC13 and dG11···dC12 base pairs and hydrogen bonding interaction between the nitrogen atom of acridine ring and the carboxylate group of Asp216 of GLI (Fig. 8c). The analysis of DNA double helical geometry led to unveiling of some interesting facts. The basic geometry of standard right handed helical DNA should have inter base pair distance of ~3.4 Å. The intercalating agents are known to cause distortion of DNA geometry and exert DNA damaging effects. Similar observations were also noted in the equilibrated structure. In the DNA-GLI-QC ternary complex this distance is increased from 3.4 Å (supplementary Fig. S8a) to 7.0 Å (supplementary Fig. S8b) so as to accommodate the QC acridine ring. The production run of 20 ns was analyzed for the stability of the system. The 20 ns trajectory analysis for the backbone RMSD, atomic fluctuation and the B-factor supported that the system has attained stabilized state after 10 ns (supplementary Fig. S9). The binding free energy change was calculated for DNA and GLI binding in binary and ternary complexes over the last 1 ns trajectory (after 20 ns production run) (Table S1). The results show that binary complex (binding free energy −218.62 ± 13.53 kcal/mol) is more stable as compared to the ternary complex (binding free energy −124.09 ± 13.85 kcal/mol) indicating the destabilizing effect of QC on DNA-GLI complex.

To further evaluate the intermolecular interaction between DNA and GLI in the binary and ternary complexes, hydrogen bond occupancies for interface residues were analyzed after molecular dynamics simulation (20 ns) using Visual Molecular Dynamics (VMD) package over the last 1 ns trajectory^34^. It was observed that in the binary complex, more hydrogen bonds were present between DNA and GLI as compared to the ternary complex (Fig. 8d). Some of the hydrogen bonds were weakened or broken in the ternary complex (as compared to the binary complex). The analysis of secondary structure using VMD and calculation of secondary structure content using a pharmaco-informatics tool developed in house showed a difference in the binary and ternary complexes (Table S2). In the ternary complex, beta sheet and beta turn content was found to be reduced as compared to binary complex. These changes can be attributed the DNA-QC interaction and subsequent conformational changes in the GLI structure.

The computational analysis clearly established that the strength of the interaction between DNA and GLI got significantly reduced after the QC intercalation. This explains the observed reduction in the DNA-GLI binding from biological experiments. The reduced interaction between DNA and GLI can be mostly attributed to the electrostatic factors rather than the Vander Waals factors. Both EEL (electrostatic energy as calculated by the MM force field) and EGB (the electrostatic contribution to the solvation free energy calculated by GB) values are greatly influenced (Table S1 and Supplementary Fig. S10).

### NQC reduces tumor growth in *balb/c* mice

**Figure 9:**
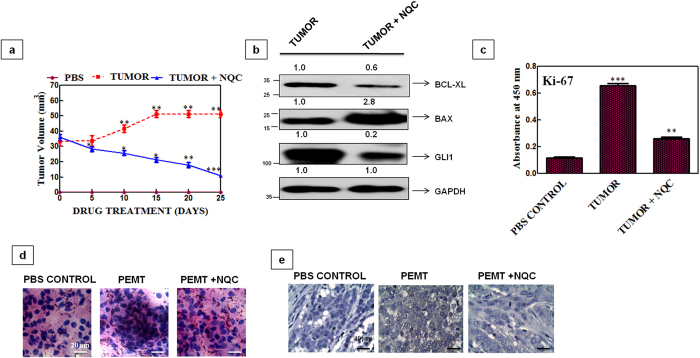
NQC caused apoptosis through HH-GLI dependent manner in PEMT-Hela (cervical CSCs) *xenograft* mice model at a dose 40 mg/kg/day (**a**) Average tumor size of NQC treated mice. (♦), (♦) and (▴) represents PBS control, tumor and NQC treated tumor *xenografts*. (**b**) Expressions of proteins in tumor lysates. Data presented here is the mean ± SD of three independent experiments. Statistical significance was determined by paired *t* test (***p < 0.001). (**c**) Expressions of proliferation marker Ki-67 in *xenografts* mice model. Data presented here is mean ± SD of three independent experiments. (**d**) H&E staining of the *xenograft* tissue section. Scale bar 20 μm. The data is the representative image of three independent experiments. (**e**) Immunohistochemical expression of GLI-1. Scale bar 40 μm. Data is the representative image of three independent experiments.

To measure the effect of NQC in tumor of *xenograft* mice, an experiment was carried out. Briefly, three groups of mice were taken. First group of mice were implanted with PBS (vehicle) and were used as control and other two groups of mice were implanted with PEMT cells subcutaneously into the left flank of animal bilaterally. The animals were kept for 25 days for the development of tumor. When a measurable amount (approximately 40 mm^3^) of tumor was formed, then they were treated with NQC by oral administration at a dose of 40 mg/Kg/day and kept for another 25 days. A reduction in the tumor volume (approximately 10 mm^3^) was noticed after end of the NQC treatment in comparison to untreated mice (Fig. 9a). Then tumors were collected and tissue samples were processed for western blotting, H&E and IHC staining. Increased BAX/BCL-xL ration and decreased GLI1 expression in NQC treated lysates proved that NQC caused apoptosis though HH-GLI dependent manner (Fig. 9b). Interestingly, the expression of Ki-67 was decreased in the serum sample of NQC treated mice in comparison to untreated tumor (Fig. 9c). H&E staining results revealed small, condensed and uniform nuclei in NQC treated tumor tissue in comparison to PBS or NQC untreated mice (Fig. 9d). IHC data showed a lower expression of GLI 1 in NQC treated mice in compare to NQC untreated mice (Fig. 9e).

## Discussion

Metastasis and drug resistance are the two major challenges for cancer therapy. HH-GLI is one of the important signaling pathways for the aggressiveness of cancer stem cell growth, metastasis, and angiogenesis. Chemotherapy is the only option after surgery and radiation to treat the cancer. Although multiple small molecule inhibitors against HH-GLI signaling are tested in multiple *in vitro* and *in vivo* systems and some of them are in clinical trial but nothing is available in clinic^29^. Most of the drugs have potential to kill the cancer cells by inhibiting the components of HH-GLI axis in cancer cells but not in CSC. Due to high drug efflux, DNA repair and self renewable capabilities of CSC, it often escapes from the toxic effect of drug and survives. As a result, conventional chemotherapeutic agents are unable to kill the CSC. Thus, it demands a search for specific small molecule inhibitors against CSC.

QC is a well documented anti-cancer agent that prevents the cancer cells growth in varieties of cancer but suffers from serious limitation in clinic due to its low pharmacokinetics profile and causes immunological complication^13^,^19^,^20^. Here, using a nano formulated QC, we have for the first time shown that NQC is more cytotoxic in cancer cells and cancer stem cells than normal epithelial cells. NQC also have potential to cause apoptosis in *xenograft* tumor *in vivo*. Interestingly, the cytotoxic ability of NQC is much higher than QC as low concentration of NQC is needed to cause equal amount of cell death than QC.

Next, we examined the mechanism of increasing the cancer cell killing activity of NQC. Nano form of QC (100 nm diameter, PDI- 0.127) makes it water soluble which may increase its bioavailability. Highly positive zeta potential (ξ+2.380) facilitates higher uptake of NQC into the cells compared to QC. The electrostatic interaction between positively charged NQC particle and negatively charge cell surface receptors or nuclear import factors, leads to localized neutralization and a subsequent bending of the membrane, favoring endocytosis, resulting in easy penetration into the cell^35^. Hence, more accumulated QC inside the cells caused more apoptosis.

GLI group of transcription factor remain as a major attributor of this signaling pathway and helps in HH-GLI dependant cell survival^28^. Therefore, development of a potent inhibitor of GLI1 became the prime importance. Reduction in the expression of GLI1 and other HH-GLI components along with its downstream targets (c-MYC, CYCLIN D1) and un-alteration of these components after GLI1 knock down confirmed that NQC might target GLI1 for the inhibition of HH-GLI cascade (Fig. 4). No significant alteration in the sub-G_1_ population and GLI1 promoter activity in GLI1-KD cells confirmed the involvement of GLI1 in NQC mediated apoptosis through HH-GLI cascade.

Available literature on CSCs suggests that apart from generating heterogeneous lineages of cells, CSCs are responsible for drug resistance, metastasis and relapse of cancer. Hence targeting CSCs with drug has become crucial for the treatment of cancer. To study the effect of NQC on cervical CSCs, we isolated cervical cancer stem cells from HeLa using serum deprivation and hypoxic condition (Fig. 5). Reduced expressions of representative markers e.g. E-cadherin in EMT and spheres while increased expression of Vimentin in spheres along with higher CD-49f confirmed the validity of CSC model (EMT, spheres, PEMT). Down regulation of HH-GLI components including GLI1, increased apoptosis, reduction of GLI1 luciferase activity in PEMT and no significant change in the sub G1 population, relative GLI luciferase activity in GLI PEMT- KD cells confirmed that NQC inhibits HH–GLI signaling in cervical CSCs. Reduction of tumor size and GLI1 expression in tumor lysates in *xenograft* mice also showed NQC mediated apoptosis is GLI1dependent.

As reported earlier that GANT 61 inhibits HH-GLI by binding to zinc finger 2 and 3 of GLI protein but not to the consensus GLI binding site in the nucleus^33^. Using *in vitro* reconstitution, *in silico* and cell based assay, we showed that QC specifically binds to GLI-DNA complex. An *in silico* experiment has been carried out which inferred that QC get inserted into the GLI-DNA complex by forming π-π interaction with 5′GACCACCCA3′ responsible for GLI-DNA complex formation which resulted in GLI destabilization and inhibition of GLI dependent cell survival. The pose obtained after molecular docking does not correspond to intercalating action of QC because the distance between two GC base pairs is in the order of 3.4 Å in the macromolecular complex. This prompted us to make efforts to reposition the QC so as to create necessary space for intercalation. Subsequently, molecular dynamics simulations optimized the geometry (the inter-base pair distance which increased from 3.4 Å to 7.0 Å; supplementary Fig. S8a,b) so as to comfortably accommodate QC between the two GC base pairs *i.e.* dG10···dC13 and dG11···dC12. The intercalating agents are known to cause distortion of DNA geometry and exert DNA damaging effects. So, the result obtained from the computational analysis confirmed that the interaction between DNA and GLI1 got reduced.

The present study showed that apoptosis occurred in cervical cancer cells and CSC by NQC was mediated through inhibition of HH-GLI cascade. NQC inhibits the HH-GLI signaling by binding to consensus sequence of GLI1 in GLI-DNA complex and destabilises it.

## Materials & Methods

### Cell culture and chemicals

The human cervical (HeLa, SiHa, ME-180), colon (HCT-116), breast (MCF-7, MDA-MB 231), kidney cancer (HEK-293) and kidney normal epithelial, VERO cells, were purchased from NCCS, Pune, India, and maintained in DMEM with 1% antibiotic (100 units of penicillin and 10 mg/ml of streptomycin in 0.9% normal saline and 10% FBS (HIMEDIA, India). MCF-10A cells were grown in DMEM/F-12, medium supplemented with 10% FBS, 100 U/ml of penicillin, 100 mg/ml of streptomycin, 0.5 mg/ml of hydrocortisone, 100 ng/ml of cholera toxin, 10 mg/ml of insulin, 10 ng/ml of EGF and 1% (w/v) of L-glutamine in a humidified CO_2_ incubator in 5% CO_2_ in 37 °C. MCF-10-ATr is a transformed cell line developed from MCF-10A by continuous exposure to cigarette smoke condensate by our group and was grown according to protocol mentioned earlier^36^. PLGA (Lactide to glycolide ratio, 50:50; MW 40,000–50,000 g mol^−1^), Poly-L-lysine,QC, PVA were procured from Sigma Chemicals Ltd. (St Louis, MO, USA). 100 mM stock of QC was prepared in DMSO of cell culture grade and stored in −20 °C. Antibodies used in the experiments were procured from Cell Signaling Technology (MA, USA) and Abcam (Cambridge, United Kingdom).

## Preparation of NQC and characterization

### Synthesis of NQC

PLGA capped NQC were prepared from QC and PLGA (50:50 lactide-glycolide ratio; inherent viscosity 1.32 dL/g in at 30 °C) using modified nano-precipitation technique^37^. In brief, 90 mg of PLGA was dissolved in 10 mL of acetone over a period of 3 h and 1 mg of QC was added to get a uniform PLGA-QC solution. This solution was drop wise added to 20 mL of aqueous solution containing 2% (wt./v) PVA (M.W. 30,000–70,000) and 10 mg of Poly-L-Lysine (PLL) (M.W. 30,000–70,000), under constant stirring at 800 rpm over a period of 10 min on a magnetic stirrer operated at 800 rpm. Within a few minutes precipitation was observed in the aqueous layer. This suspension was stirred at room temperature for 24 h for complete evaporation of acetone. Unentrapped QC was removed by centrifugation at 5,000 rpm for 20 min. PLGA-NPs with entrapped QC were recovered by ultracentrifugation (Beckman Coulter Ultracentrifuge Optima L-90 K, USA) at 35, 000 rpm for 15 min at 4 °C followed by overnight lyophilization (Free Zone Bench top Freeze Dry System, Lanconco, MO, USA). Lyophilized NQC was dissolved in water for further experimentation.

## Characterization of NQC

### UV-Visible Spectroscopy

Synthesis and stability of the formulated NQC was observed at regular intervals based on the Surface Plasmon Vibrations in a UV-Vis spectrophotometer at 420 nm (EPOCH™ Multi-Volume Spectrophotometer System, Biotek instruments Inc., Mumbai, India)^38^. The stability of NQC was checked in the UV-Vis spectrum ranging from 200–900 nm. NQC showed a characteristic peak at 420 nm with a particle size of 100 nm in comparison to other components involved in the formulation.

### TEM analysis

NQC was also evaluated for size by transmission electron microscopy (JEOL-JEM 2100, 1.4 Angstrom Unit, Tokyo, Japan) in a method described earlier^38^. Briefly, a drop of the NQC suspension was placed over the carbon coated copper TEM grid (150 mesh, Ted PELLA Inc, Rodding, CA) and allowed to dry. Then, it was shifted to the specimen holder and images were visualized at 120 KV under the microscope.

### DLS & Zeta potential analysis

The particle size and size distribution were elucidated by ZETASIZER (3000, Malvern, UK) as described earlier^38^. The average of hydrodynamic particle size was expressed as the value of Z-average size ±S.D. from three replicated samples. Additionally, zeta potential data was recorded by ZETASIZER (3000, Malvern, UK) to know the surface charge distribution and expressed as mV.

### FTIR analysis

Chemical integrity of the drug and the polymer matrix was investigated using FTIR spectra (PerkinElmer Model Spectrum 1, PerkinElmer, MA, USA). Samples were crushed with potassium bromide (KBr, Sigma-Aldrich) as described earlier to get the pellets by applying a pressure of 300 kg/cm2^38^. FTIR spectra of NQC and QC were scanned in the range between 4000 and 400 cm^−1^.

### XRD analysis

NQC along with QC were subjected to XRD analysis to elucidate the face centered cubic crystalline nature of the formulated NPs. Briefly, the lyophilized form of the synthesized NQC and QC were coated on XRD grid and the spectra were recorded using powder X-ray diffractometer (D8 Advance Powder XRD, Bruker, USA). The diffracted intensities were recorded from 5 to 80° at 2θ angles^38^.

### Uptake analysis of NQC and QC

Uptake analysis of QC and NQC was measured by FACS analysis as described earlier^39^. Briefly1×10^6^ cells were seeded in a 6 well cell culture plate and incubated for 24 h. Cells were exposed to increasing concentrations of NQC as well as QC and incubated for 6 h. After incubation, supernatant was removed and cells were rinsed with 1X PBS. Then, cells were harvested by trypsinization and processed for the uptake study with the flow cytometer (FACS Canto II, Becton & Dickinson, CA, USA). Pacific blue filter was used to detect the fluorescence and 10,000 events were recorded for each sample. Fluorescence was expressed as Mean Fluorescence Intensity (MFI) by comparing the increase in fluorescence of the treated sample with that of the untreated one.

### MTT assay

The anchorage dependant cell viability of NQC and QC was measured using MTT assay as described earlier^40^. Briefly, exponentially growing cells (8000–10,000 cells/well) were seeded in 96 well flat bottom tissue culture plates and allowed to grow for 70–80% confluence. Cells were treated with increasing concentrations of NQC and QC for 48 h before harvest. After washing with 1X PBS 0.05% MTT was added to each well and incubated in 37 °C for 5 h for the formation of formazan crystals. The crystal was dissolved by the addition of 100 μL of 0.2% NP-40 detergent after incubating in dark for 1 h. The colour intensity was measured spectrophotometrically at 570 nm by using microplate reader (Mithras LB 940, Berthold, Germany). The data obtained were presented graphically as percent viability against concentrations. Each data point was calculated in triplicate and all the assays were performed at least thrice.

### Clonogenic assay

The colony forming ability of cells after exposure to NQC and QC was measured using a well-defined assay described earlier^40^. HeLa cells (500/well) in 70–80% confluent were treated with NQC and QC for 48 h. At the end of incubation the media was removed and replaced with the fresh media and then allowed to grow for 6-8 doublings. Finally, the plates were washed with 1XPBS and air dried followed by staining with 0.2% crystal violet for 1 h. The colonies were washed repeatedly with distilled water and air dried and then counted with the help of a colony counter. The data were calculated and graphically represented as percent survival against concentrations. Each data point was calculated in triplicate and the assays were performed at least three times.

### DAPI nuclear staining

To measure apoptosis after drug treatment, DAPI (4, 6-diamidino-2-phenylindole) nuclear staining experiment was performed according to the protocol described earlier^41^. 80–90% confluent HeLa cells (5×10^3 ^cells/well) were treated with various concentrations of NQC and QC for 48 h. After treatment, media was aspirated and the cells were washed with 1X PBS and fixed with acetone: methanol (1:1) and kept in −20 °C for 20 min. Fixed cells were washed with 1X PBS followed by staining with DAPI dye and incubated in dark in 4 °C for 30 min. Excess DAPI were washed with 1XPBS and observed under fluorescence microscope (Nikon, Japan) in 40X magnification.

### Analysis of cell cycle regulation by FACS

In order to check apoptosis and regulation of cell cycle after NQC and QC treatment, a FACS analysis was carried out as described earlier^42^. Briefly, 1×10^5^ cells were seeded in two separate 6 well tissue culture plates and were treated with increasing concentrations of NQC and QC for 48 h. Cells were harvested by trypsinization and fixed with ice chilled 70% ethanol and then incubated over night at −20 °C. Then, cells were washed and stained with 0.1 mL of 50 μg/mL Propidium Iodide (PI) containing 0.05% RNase and then incubated in dark at 37 °C. Cells were sorted by FACS (FACS CANTO II, Becton & Dickinson, CA, USA) with an event count of 10,000 events per sample. Analysis of data was done by FACS diva software.

### Western Blot

To check the expressions of proteins, western blot analysis was carried out according the protocol described earlier^43^. For this, cells(1×10^6^) were plated in a 60 mm cell culture discs and were grown for 24 h. Cells were exposed to NQC and QC for 48 h and were harvested by scraping. The pellet was resuspended in RIPA (50 mMtris, 150 mMNaCl, 0.5 mMdeoxycholate, 1% NP-40, 0.1% SDS, 1 mM Na_3_VO_4_, 5 mM EDTA, 1 mM PMSF, 2 mMDTT, 10 mM β-glycerophosphate, 50 mMNaF, 0.5% triton X-100, protease inhibitor cocktail) lysis buffer for 45 min at 4 °C. After centrifugation at 14,000 rpm for 10 in at 4 °C, the supernatant was collected. Approximately, 100 μg of proteins were separated on SDS-PAGE and transferred onto a PVDF membrane. Then, the membrane was probed with specific antibody according to antibody manufacturer’s protocol. The number above each blot represents relative fold change compared to untreated control measured by densitometry.

### Knockdown of GLI1 in HeLa cells

Wild type GLI1gene was knocked down from HeLa cell line using SiRNA specific to GLI1 (CAT#SC-37911, Santacruz Biotechnology) according to protocol described earlier^43^. Briefly, cells (1×10^5^) were grown in 60 mm tissue culture discs to 70% confluency. Cells were transiently transfected with either 4 μg of SiRNA specific to GLI1 or scrambled SiRNA using transfection reagent Lipofectamine 2000®. After 8 h,media was replaced with fresh media and grown for another 24 h. Then, cells were treated with NQC for 24 h and processed for experimentation.

### Luciferase based reporter assay

To measure the promoter activity of GLI1, a luciferase based reporter assay was performed according to the protocol described earlier^42^. Cells (1×10^5^) were grown in a 60 mm tissue culture discs to 70% confluency. Then, were transfected with 2.0 μg GLI1 luciferase plasmid (PGL2 GLI Luc) along with 0.5 μg β-gal plasmid using Lipofectamine 2000® (Invitrogen). After 8 h,media was replaced with the fresh media and grown for another12 h. Cells were treated with NQC for 24 h before harvest. Luciferase activity in lysates were measured using microplate reader (Multimode ELISA Reader, Berthold, Germany) and β-gal activity was measured to normalise the transfection efficiency. Relative luciferase activity of GLI1 was calculated from triplicate experiments, and data were plotted against concentrations of NQC. Similar set of luciferase assay was performed with GLI1 knockdown cells.

### m-RNA expression of GLI1

1 × 10^5^ seeded HeLa cells were treated with increasing concentrations of NQC for 48 h. Total RNA was isolated using GeneJET RNA Purification Kit (Cat No#K0732, Fermentas, USA). c-DNA was synthesized from the harvested RNA using revert AID first strand cDNA synthesis kit (CAT#K1622, Fermentas, USA). Amplification was done by PCR. Sequence for primers is as follows; forward: 5′TCCCTGCCTGGCCTTATG3′ and reverse: 5′ATCAAAGTCCAGGCAAGGC3′. All the primers were purchased from Integrated DNA Technologies, USA. Samples were separated on 0.9% agarose gel and images were captured under UV and band intensity was measured by densitometry. The numerical value above each panel represented the relative fold changes of band intensity (measured by densitometer) in comparison to untreated cells.

### Culture and establishment of cancer stem cell (CSC)

Trypsinized HeLa cells were plated at a cell density of 1000 cells/ml in serum free media containing 10 ng/mL of basic fibroblast growth factor, 20 ng/mL EGF, 5 μg/mL of insulin and 0.4% BSA. Hypoxic environment was maintained using 200 μM CoCl_2_^44^. In this condition, cells grew as adherent monolayer and known as quiescent phase (EMT). The quiescent cells were grown in ultra low attachment plate and allowed to grow for 7–15 days. Some of the cells started floating as visible cluster of spheroids known as cervix spheres. The spheroids were collected and centrifuged at 850 rpm for 5 min followed by dissociation using Trypsin-EDTA and mechanically by Pasteur pipette^45^. Single cell suspension of spheroids were cultured in normal growth medium containing 10% FBS. They were grown as adherent monolayer representing PEMT.

### Indirect and Sandwich ELISA

Detection of soluble markers of CSCs was done by Indirect ELISA as per the protocol described earlier^46^ and detection of proliferation marker Ki-67 was done by sandwich ELISA. Briefly, for indirect ELISA the protein antigen was mixed with coupling buffer, coated on to 96 well microplate and incubated overnight at 4 °C. After washing with wash buffer, the wells were blocked with blocking solution (1% BSA in PBST) and incubated at RT for 30 min. Then, primary antibody (anti-CD-49f) was added, incubated for 2 h at RT. After washing with PBST, the wells were then incubated with HRP conjugated secondary antibody for 1 h. It was then washed twice with PBST. Then, the substrate solution i.e. 2,2′-azinobis-(3-ethylbenzthiazoline-6-sulphonic acid) was added and the absorbance of the coloured product formed was detected at 405 nm using microplate reader. For sandwich ELISA the precoated (Ki-67) plates were incubated with sample with biotin conjugated antibody specific to Ki-67 protein. Then, avidin conjugated Horseradish peroxidase (HRP) was added to each microtiter well. Then, TMB substrate was added and the enzyme substrate reaction was terminated by addition of stop solution and the absorbance was measured at 450 nm.

### Annexin V-FITC/PI dual staining for apoptosis measurement

Annexin V-FITC/PI dual staining is used as a measure of apoptosis. For this assay, cells were seeded in a 6 well cell culture plate at a density of 1×10^5^ cells per well. Cells were treated with different concentrations of NQC for 48 h. Then, the cells were harvested by trypsinization and washed twice with 1X PBS. Cells were resuspended in 1X binding buffer and stained with Annexin V-FITC and PI detection kit (Sigma). It was then incubated in dark for 10 min and analyzed using FACS. 10,000 cells per sample were acquired in PE and FITC channel.

### Molecular docking studies

Maestro 9.3 software package was used to perform molecular docking studies^47^. For this purpose, the 3D structure of QC was built in the Maestro 9.3. The ligand preparation module of Maestro 9.3 was used to generate various possible ionization states of QC at physiological pH (7.3 ± 2.0). The crystal structure of GLI-DNA complex (PDB ID: 2GLI; resolution 2.60 Å) was prepared for the molecular docking studies using Protein Preparation Wizard^48^. Missing hydrogens were added, right bond order was assigned and water was removed. Conformational and tautomeric states of amino acids were optimized. The receptor interaction grid was generated at the various possible binding sites in the GLI-DNA complex using the Receptor Grid Preparation wizard. Molecular docking studies were performed using Glide 5.7 module to generate the ternary complex of DNA, GLI and QC (supplementary Fig. 7)^49^. The QC is reported to intercalate preferentially between GC base pairs in DNA^8^. To achieve this intercalating pose, manual repositioning of the QC was carried out between the GC base pairs nearest to the docked pose *i.e.* dG10···dC13 and dG11···dC12. This complex was further optimized using molecular dynamics simulation (see supporting information for detailed description).

### Molecular dynamics simulations (MD simulations)

Molecular dynamics simulations were performed on the DNA-GLI binary complex and DNA-GLI-QC ternary complex (generated after the manual intercalation of the docked QC) using AMBER 11 package^50^. The partial atomic charges for QC were derived using AM1-BCC method of *antechamber* module of AMBER^51^*) in *antechamber* module of AMBER^51^. For the preparation of ligands and protein, General Amber Force Field (GAFF) and Amber ff99SB force field were used respectively^52^,^53^. Systems were solvated using TIP3P water, with solvation box extended to 20 Å in each direction of the solute forming an octahedral box^54^. The initial minimization of the systems was followed by gradual heating from 0 to 300 K, with system restraint of 2 kcal/mol/Å^2^ (NVT ensemble) for 50 ps. The density equilibration (NPT ensemble) of the protein was carried out in three phases *i.e.* 50 ps equilibration with weak restraint of 2 kcal/mol/Å^2^, followed by 50 ps with restraint of 1 kcal/mol/Å^2^ and then unrestrained density equilibration for 100 ps. The constant pressure equilibration (NPT) of 1 ns was performed at 300 K and 1 atm pressure (pressure relaxation time of 2.0 ps). Finally, production run for 20 ns was performed under NPT ensemble. The long-range electrostatic interactions were treated with the Particle-Mesh Ewald (PME) method^55^. For the calculation of relative binding free energy for the DNA and GLI, Molecular Mechanics-Generalized Born Surface Area (MM-GBSA) method available in Amber11 package was used^56^. The calculations were performed on the last 1 ns trajectories obtained from MD simulations of protein-ligand complexes to ensure good conformational sampling and reliable binding free energy calculations. The hydrogen bond occupancies were analyzed using Visual Molecular Dynamics Package (VMD)^34^.

### Measurement of GLI1 level in GLI-DNA complex in nuclear lysate of NQC treated PEMT-CSC cells

Level of GLI1 in nuclear lysates of PEMT-CSC cells in GLI-DNA complex was measured using Dynal-M270 streptavidin-magnetic beads according to protocol described earlier^57^. Briefly, cells were treated with different concentrations of NQC and then nuclear lysates were prepared using NE-PER kit (PIERCE Biotechnology, Cat. No.#78833). The beads were blocked with 5% BSA and it was washed four-times with buffer I (10 mMTris-HCl, pH 7.6, 1 M NaCl and 1 mM EDTA) using a magnetic separator rack (Dynal, Invitrogen, Carlsbad, CA). Then, the beads were incubated with an oligonucleotide sequence (5′GACCACCCA3′) (custom made from Biotech Desk, Hyderabad, India) at constant agitation for 30 min at RT in buffer I. After washing, the beads were resuspended in buffer II (30 mM HEPES, pH 7.5, 30 mMKCl, 8 mM MgCl_2_, 5% glycerol, 0.2 mM ATP, 0.5 mM DTT). Then, the beads were incubated in binding buffer containing 30 mM HEPES, pH 7.5, 30 mMKCl, 0.5 mM DTT, 5% glycerol and nuclear extract (30 μg) of CSC cells at 37 °C for 1 h. Reactions were terminated by quickly removing the GLI-DNA complex beads from unbound proteins by applying the magnetic field using the separator rack. Then, the beads were washed with buffer II. The washed beads containing active GLI proteins were resuspended in the reaction buffer (30 mMHepes, pH 7.5; 30 mMKCl, 8 mM MgCl2, 1 mM DTT, 0.01% (v/v) Nonidet P-40) and directly used for measuring the activities of GLI1 by western blot.

### Development of tumor *xenograft* in balb/c mice

*In vivo* animal experiment was carried out according to the protocol described earlier^36^. 6 weeks old female *balb/c* mice were maintained in proper light /dark cycle of 12/12 h at Institute of Life Sciences, Bhubaneswar, India. All the animal work and the experimental protocol were approved by institutional animal ethical committee (IAEC, Institute of Life Sciences, Bhubaneswar, India). All the experiments were carried out in accordance with approved guidelines of the institute. 1×10^7^ HeLa-PEMT cells in 200 μl freshly prepared sterile PBS were injected subcutaneously into the flank of animal bilaterally. Three groups of mice (6 mice in each group) were taken for the study. One group of mice were implanted with PBS (control). Other two sets of mice were implanted with HeLa-PEMT cells for the development of tumor. Tumor growth was measured twice a week. After the end of 25 days when a measurable amount of tumor was noticed, then, 100 μl of NQC (dissolved in PBS) was administered in one group by oral route in order to get a dose of 40 mg/kg/day which will be equivalent to 195 mg in a 60 kg human adult^36^. Tumor dimension were measured by slide calliper and tumor volume was calculated using the formula: (W^2^ × L)/2 (W is width and L is length) and growth curves were drawn. At the end of 25 days, the mice were euthanized and the tumor tissues were collected and processed for protein extractions and western blotting.

### H&E staining and Immunohistochemistry

For H & E and IHC staining frozen tissues were cut in a section of 5 μm using Shandon cryotome FSE (Thermo scientific) mounted on superfrost® plus slides (Thermo Scientific) at −30 °C^41^. For H&E slides were rehydrated by step by immersing the slides in different percent (100% to 50%) of alcohol. Dried slides were dipped into hematoxylin followed by eosin (H&E). Then slides were dehydrated by immersing the slides in different percent of alcohol starting from 50% to 100% and finally incubated in xylene for 2 minutes. Images were captured in 20× magnification using inverted microscope (Nikon, Japan). For IHC fixed slides were blocked with 100 μl blocking solution (TBS with 5% FBS) followed by immunostaining with GLI1 primary antibody and incubated overnight at 4 °C. After washing with 1× TBST slides were incubated with conjugated secondary antibody and incubated for 30–60 minutes at room temperature. Finally images were captured in 10X magnification using inverted microscope (Nikon, Japan).

### Statistical analysis

A two-tailed Student’s t-test was employed and *P < 0.05, **P < 0.005 and ***P < 0.0001 was considered to be statistically significant.

## Additional Information

**How to cite this article**: Nayak, A. *et al*. Nanoquinacrine induced apoptosis in cervical cancer stem cells through the inhibition of hedgehog-GLI1 cascade: Role of GLI-1. *Sci. Rep.* **6**, 20600; doi: 10.1038/srep20600 (2016).

## Supplementary Material

Supplementary Information
