# Supplementary material for: Nanoquinacrine induced apoptosis in cervical cancer stem cells through the inhibition of hedgehog-GLI1 cascade: Role of GLI-1
Source: Sci Rep. 2016 Feb 5;6:20600. doi: 10.1038/srep20600 (PMC4742869; doi:10.1038/srep20600)
Supplement: Supplementary Information [file srep20600-s1.pdf]

Nanoquinacrine induced apoptosis in cervical cancer stem cells through the inhibition of hedgehog-GLI1 cascade: Role of GLI-1

Anmada Nayak[1#], Shakti Ranjan Satapathy[1#], Dipon Das[1], Sumit Siddharth[1], Neha Tripathi[2], Prasad V Bharatam[2,3], ChanakyaNath Kundu[1*]

[1]Cancer Biology Division, KIIT School of Biotechnology, KIIT University, Campus-11, Patia, Bhubaneswar, Orissa, 751024, India.

[2]Department of Pharmacoinformatics, National Institute of Pharmaceutical Education and Research (NIPER), Sector 67, S.A.S. Nagar, Mohali, Punjab 160062, India

[3]Department of Medicinal Chemistry, National Institute of Pharmaceutical Education and Research (NIPER), Sector 67, S.A.S. Nagar, Mohali, Punjab 160062, India

# **Equal contribution**

Running title: Inhibition of HH-GLI cascade in cervical CSC by NQC

Materials & Methods

Western Blot

Described in the main document.

Caspase-3 Immunofluorescence

The expression of caspase 3 on cervical cancer stem cell was done by immunofluorescence assay. For this assay briefly, 10,000 PEMT cells were seeded in the well of culture plates on the surface of cover slip and allowed to grow for 24 h [1]. Then the cells were treated with different concentrations of NQC for 48 h. Then cells were washed with 1X PBS and fixed with fixer acetone: methanol (1:1) at -20ºC for 20 min. After 20 min cells were blocked with 2% BSA and 0.2% triton X-100 in PBS for 20 min in 37°C. After that cells were incubated with primary antibody for 2 h. Then the cells were washed with 1X PBS and incubated with secondary antibody conjugated with TRITC and incubated for 1 h. After that cells were washed with 1X PBS and stained with DAPI for 10 min. Again cells were washed and images were taken using fluorescence microscope (AMG Evos Fluorescence Microscope, Thermo Fisher Scientific, MA, USA).

Proteasome mediated inhibition study

To study the impact of proteasome meditated degradation of GLI1 by NQC in PEMT cells, approximately 1×10^6 cells were seeded in a 6 well cell culture plate. After 80% confluency cells were pre-treated with MG-132 (5µM) for 3h followed by NQC treatment for 48 h prior to harvest [2]. Then whole cell lysates were prepared and processed for western blot experiment.

UV-Vis spectroscopic study

To check the drug -DNA interaction we have used UV-VIS spectrophotometric based assay. For this a fixed concentration of DNA (50µg/mL) was incubated to varied concentrations of NQC (upto 1 µM) in solution of 50 mM Tris-HCl/NaCl (pH 7.5) for 1h at 37ºC [1]. We have used two types of DNA i.e. wild type APC which do not contain GLI- DNA binding consensus sequence and PGL2GLI-LUC plasmid (which contain the GLI-DNA binding consensus sequence). After end of the incubation an absorption spectra was taken within wavelength 200-320 nm.

Molecular docking studies

Protein Structure Preparation: The 3 dimensional crystal structure of GLI-DNA complex is available in the Protein Data Bank (PDB ID: 2GLI) at a resolution of 2.60 Å [3]. The GLI-DNA complex, GLI and DNA structures were further prepared for the molecular modeling studies using Protein Preparation Wizard module of Maestro 9.3 package [4]. The missing hydrogens were added and right bond order was assigned. For optimizing the orientations of hydroxy group (in Ser, Thr and Tyr), amino group (in Asn and Gln) and ionization state (His), protassign utility and impref utility of the Protein Preparation Wizard were used.

Receptor Interaction Grid Generation and Molecular Docking: The receptor interaction grid was generated at the various possible binding sites in the macromolecular structures (GLI-DNA complex, DNA and GLI) using the Receptor Grid Preparation wizard of Maestro 9.3. The considered region for this purpose include DNA-binding motif in GLI *i.e.* residue Phe174-Pro199 (grid center: -18.05, -4.07, 10.84), GLI-binding motif in DNA (grid center: -15.48, 12.45, 11.03), the GANT61 binding site in GLI (grid center: -29.38, 0.60, -6.43) and at the center of DNA-binding and GLI-binding motif in GLI-DNA complex (grid center: -17.04, 2.45, 10.91) [5]. Each grid box was extended up to 10 Å as the inner box and 20 Å as the outer box covering the binding site cavity completely in each case. The prepared ligand QC was docked in the generated grids in GLI-DNA complex, DNA and GLI structures using Glide docking module (Glide. version 5.7 ed.; Schrödinger, LLC: New York, 2012) of Maestro. Each of the poses were analysed for molecular recognition interactions and Glide docking score [6]. The generated DNA-GLI-QC ternary complex was found to be satisfactory in terms of interactions and score. The QC is a DNA intercalator occupying the regions having GC base pair. Therefore, a manual intercalation of the DNA was performed between the GC base pairs present near the docked pose (dG10···dC13 and dG11···dC12). The cationic tail was facing towards the minor groove and the acridine ring was inserted at the base pair interfaces. This complex was further optimised using molecular dynamics simulation.

Results

NQC induces apoptosis in cervical cancer cell line by targeting HH-GLI axis

In order to confirm further the effect of NQC on HH-GLI pathway, protein expressions of different intermediates in HH signaling were measured in SiHa cell line. A dose dependant decrease in the expressions of oncogenic ligand SHH, oncogenic protein SMO and transcriptional activator GLI1were noted in NQC treated cells. The expressions of c-MYC and CYCLIN D1 that act immediately downstream to GLI1 also decreased in same cellular lysate (Supplementary Fig. S1).

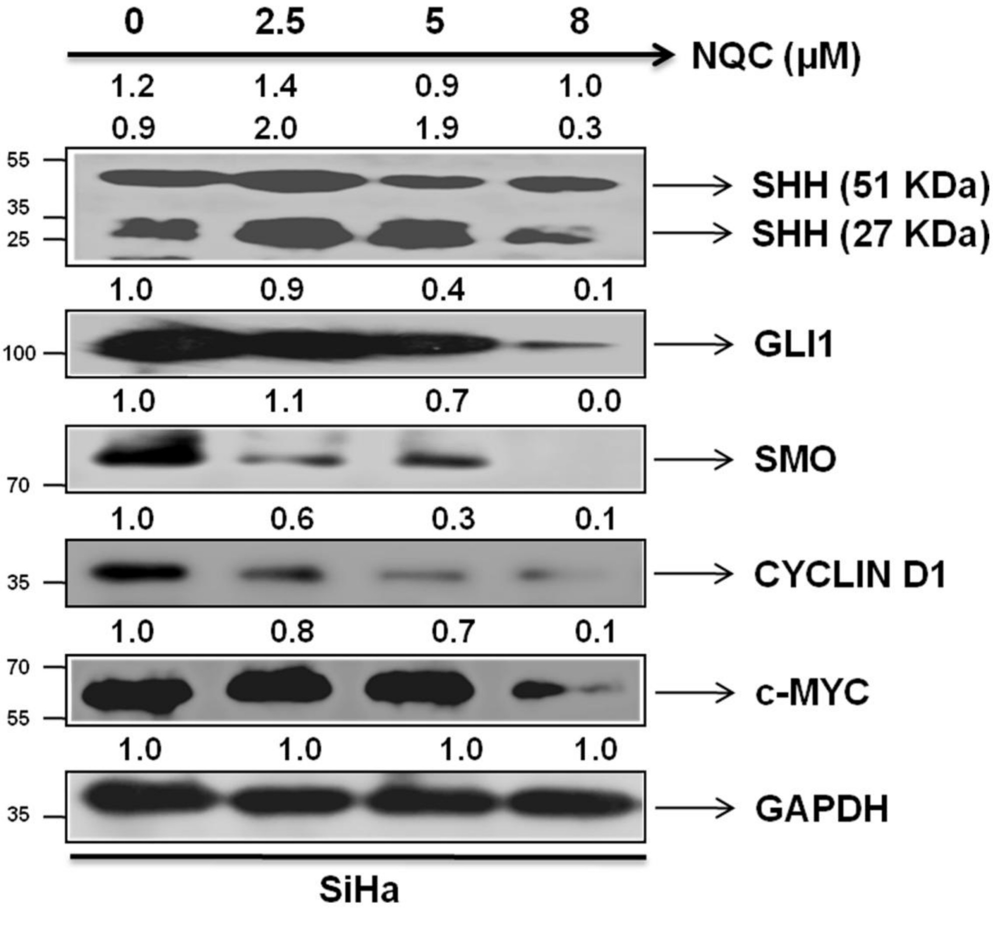

Supplementary Figure S1: Expressions of HH components in NQC treated SiHa cells. Data represented here was the replication of three different experiments. The numerical value above each panel indicates the relative fold changes of band intensity (measured by densitometer) in comparison to untreated cells. GAPDH was used as a loading control.

NQC increases the expression of caspase 3 in PEMT CSC cell line

Caspase 3 represents a marker of apoptosis. Increase in the formation of apoptotic nuclei along with dose dependent increase in the caspase 3 expression with respect to untreated control revealed that NQC caused apoptosis in PEMT cell line (Supplementary Fig.S2).

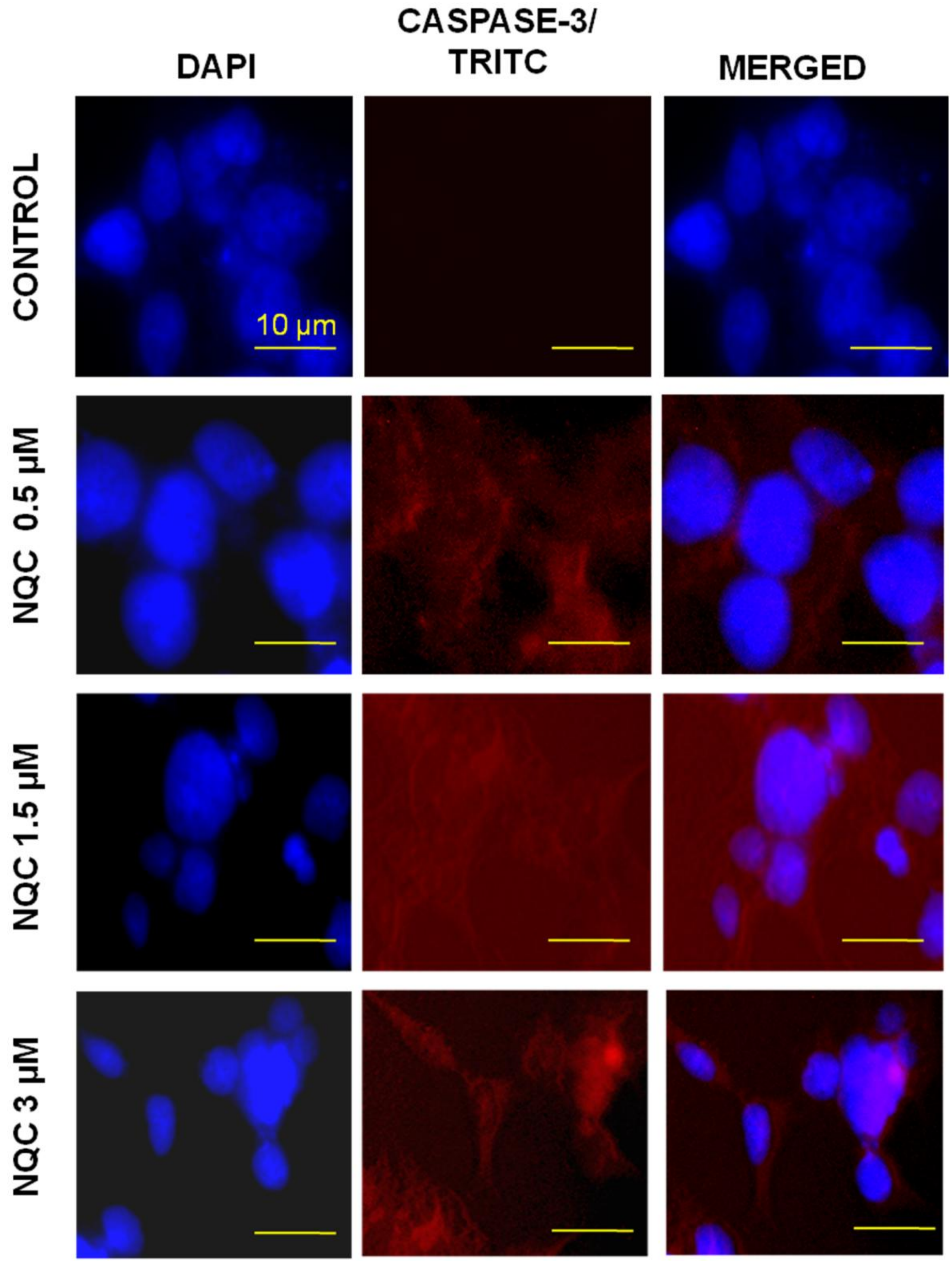

Supplementary Figure S2: Immunocytochemical analysis of apoptotic marker caspase 3 expression in PEMT cells: Immunofluorescence of caspase 3 in PEMT was carried out after

treatment with NQC for 48 h. Fixed cells were stained with caspase 3 antibody and respective TRITC conjugated AR secondary antibody and DAPI observed under fluorescence microscope (EVOS) using DAPI and red filter.

Proteasomal degradation of GLI1

The GLI1 expression was found to be decreased after NQC exposure (Figure 6b main text) in PEMT cells. In order to evaluate whether the decrease was due to proteasomal degradation of GLI1 after being destabilized from the DNA-QC-GLI1 complex, proteasomal degradation assay was carried out using proteasomal degradation inhibitor MG-132. PEMT cells were pre-treated with MG-132 and then exposed to increasing concentration of NQC. It was observed that blocking the proteasome using MG-132 restored GLI1 levels even after NQC treatment (Supplementary Fig.S3a). GAPDH was used as a loading control.

Auto-activation of GLI1

GLI1 has been previously reported to bind specifically to a consensus promoter sequence of 5'GACCACCCA3'[7]. In order to check whether GLI1 binds to the promoter sequence of the GLI1 gene and activates itself, the presence of the consensus sequence in the promoter sequence of GLI1 gene was evaluated. Supplementary Fig.S3b shows the presence of the consensus sequence 5'CGGGTGGTC3'which is complimentary to 5'GACCACCCA3'[7]. In order to check whether NQC has any specificity towards the binding of this sequence an *in vitro* DNA-drug binding assay was carried out (Supplementary Fig.S3c). In principle, pure DNA gives a peak of absorbance spectra at 260nm wavelength, but when a drug binds to it the absorbance spectra shifts left as the binding of the drug decreases the absorbance spectra [2]. pGL2-GLI1 promoter was used for the *in vitro* study. It was observed that pure DNA gives an exact spectrum of 260 nm but with increasing concentrations of NQC, there is a shift in the absorption spectra indicating the possible binding of the NQC to the DNA sequence (Supplementary Fig.S3c). Supplementary Fig. S3d gives the K_d value i.e. half saturation value of binding isotherm and was found to be 2.5×10^{-6} M^{-1}(Supplementary Fig.S3d).Supplementary Fig. S3e is the spectral analysis of NQC to a wild type APC DNA and the spectral scan indicated that NQC has no significant role in binding to APC DNA. So, this result confirms that NQC very specifically binds to the GLI DNA sequence.

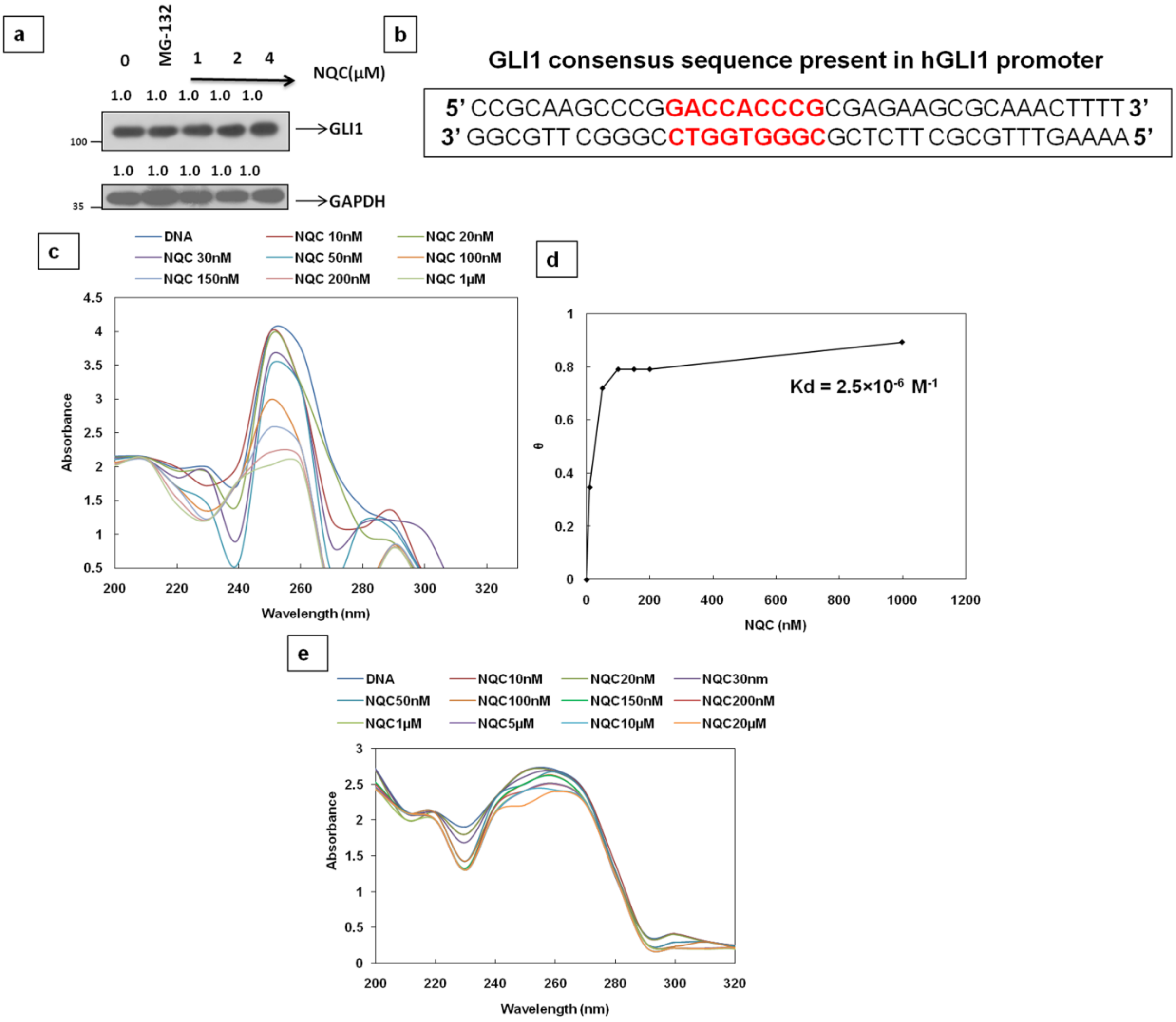

Supplementary Figure S3: (a) Expression of GLI1 protein after exposure to MG-132 followed by NQC treatment. The lower panel shows the loading control. The numerical value above each blot describes the fold change in the expression level in compare to control. **(b)** Represents the sequence of GLI1 promoter**. (c)** UV-Visible absorption spectra of NQC to PGL2-GLI1 promoter interaction**. (d)** Represents the binding constant and mode of binding of NQC to the consensus complimentary GLI binding sequence and Kd represents the bonding constant**. (e)** UV- Visible absorption spectra of NQC and APC wild type DNA interaction.

Electrostatistical analysis of QC and 2GL1 (DNA-GLI complex)

The available information on QC interaction with DNA states that the acridine ring of QC intercalates between the GC base pairs and the cationic tail interacts with the DNA backbone [8]. To understand the electrostatistical basis of DNA-GLI complexation, surface potential of GLI was

visualised using Pymol. As expected, the DNA-GLI interface is complementary in terms of surface potential *i.e.* DNA has highly electronegative sugar-phosphate backbone whereas the GLI surface at the DNA-GLI interface is highly electropositive (Supplementary Fig. S4a). This confers the high stability to the DNA-GLI complex.

The electro-statistic nature of QC surface (visualised using Jaguar module of Maestro9.3) was analysed to understand the reported facts about QC-DNA interaction [9, 10]. The QC has acridine ring which can intercalate in the GC base pair rich region. The cationic tail can interact with the DNA sugar-phosphate backbone suitably (Supplementary Fig. S4b). Due to similar electro-statistics of QC tail and GLI *i.e.* electropositive nature, GLI and QC cannot interact efficiently.

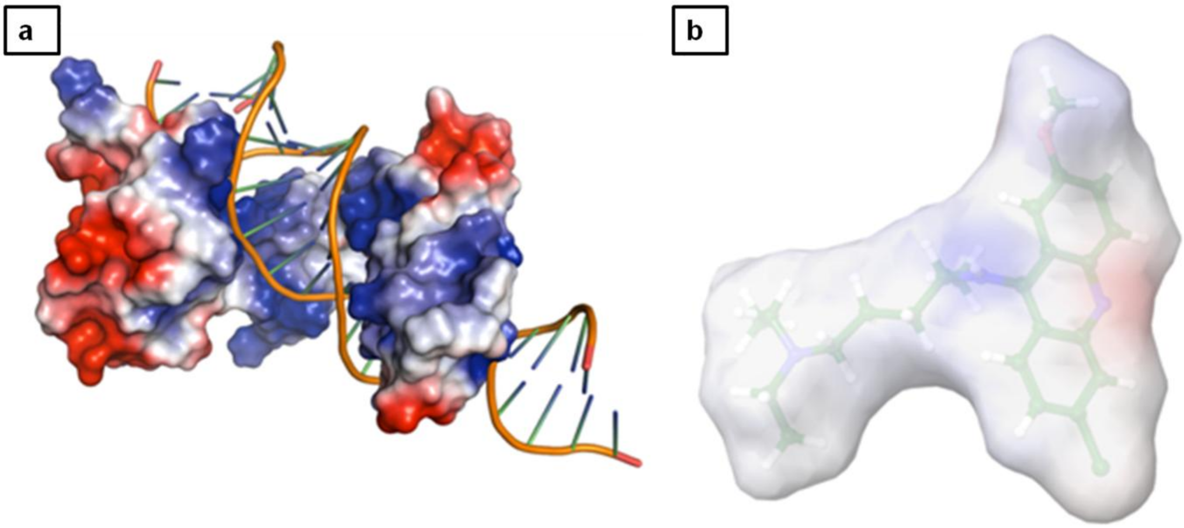

Supplementary Figure S4: Surface electrostatic potential of **(a)** DNA-GLI complex and **(b)** QC. Red colour indicates electronegative potential whereas blue colour indicates the electropositive potential.

Cavity Analysis in the DNA, GLI and GLI-DNA Complex

The 3D macromolecular structure (PDB ID: 2GLI) was analysed for the possible binding sites in the crystal structure using CastP server [11]. For this purpose, three structures were analysed *i.e.* DNA-GLI complex, DNA and GLI. The analysis revealed the possible binding sites in the macromolecular structures (Supplementary Fig.S5). The cavity volume and QC volumes were found to be complementary to the cavities in DNA-GLI binary complex. The molecular volume of QC is

291.2 Å^3 whereas the cavity volumes were 564.7 Å^3 and 440 Å^{3}in the binary complex. The cavity volume in DNA and GLI were found to be 895.3 Å^3 and1051 Å^3 respectively. These results indicate that the binding sites formed due to the complexation of DNA and GLI can be considered as the binding pocket for the QC. These sites were further targeted for the molecular docking of QC in DNA-GLI complex. Further, the molecular docking of QC in DNA and GLI were performed in the possible binding sites.

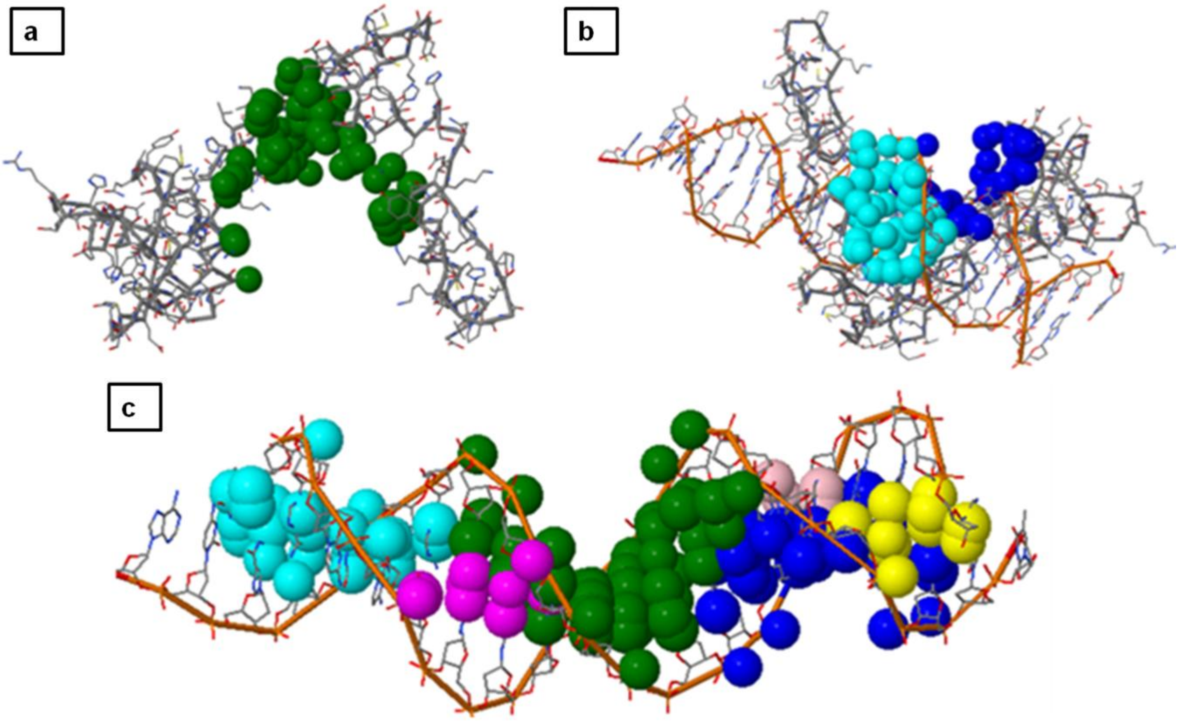

Supplementary Figure S5: Cavity analysis for the (**a**) GLI (**b**) DNA-GLI complex and (**c**) DNA.

Molecular Docking Studies

For the molecular recognition interaction analysis for DNA-GLI-QC complex, molecular docking of QC was performed in various possible sites which include the DNA-binding motif ([Y/F]JXCX3GCX3[F/Y]X5LX2HX4H[T/S]GEKP) of GLI *i.e.* residue Phe174-Pro199 (glide docking score: -3.330), the GLI-interacting GACCACCCA consensus of DNA (glide docking score: -7.278), the center of DNA-GLI-interaction motifs in GLI-DNA complex (glide docking score: -6.431) and the GANT61 binding site (glide docking score: -4.218). Finally, the results of QC docking in the DNA-GLI complex were considered for further studies.Since, our interest lies in analysing the effect

of NQC on DNA-GLI complexation; the NQC molecular docking results in DNA-GLI complex at the DNA-GLI interface was considered (glide docking score: -6.431) (Supplementary Fig.S6 and Supplementary Fig.S7a). Manual repositioning of NQC between the GC base pairs (Supplementary Fig.S7b) led to many close contacts between the GC base pairs and NQC which were removed by molecular dynamics simulation. The analysis of DNA double helical geometry led to unveiling of some interesting facts. The basic geometry of standard right handed helical DNA should have inter base pair distance of ~3.4 Å. In the DNA-GLI-NQC ternary complex this distance is increased from 3.4 Å (Supplementary Figure S8a) to 7.0 Å (Supplementary Fig. S8b) so as to accommodate the NQC acridine ring. Supplementary Fig.S9 shows the surface potential of 2GLI (the crystal structure of DNA-GLI complex. The production run of 20 ns was analyzed for the stability of the system. The 20ns trajectory analysis for the backbone RMSD, atomic fluctuation and the b-factor supported that the system has attained stabilized state after 10ns (Supplementary Figure. S9).

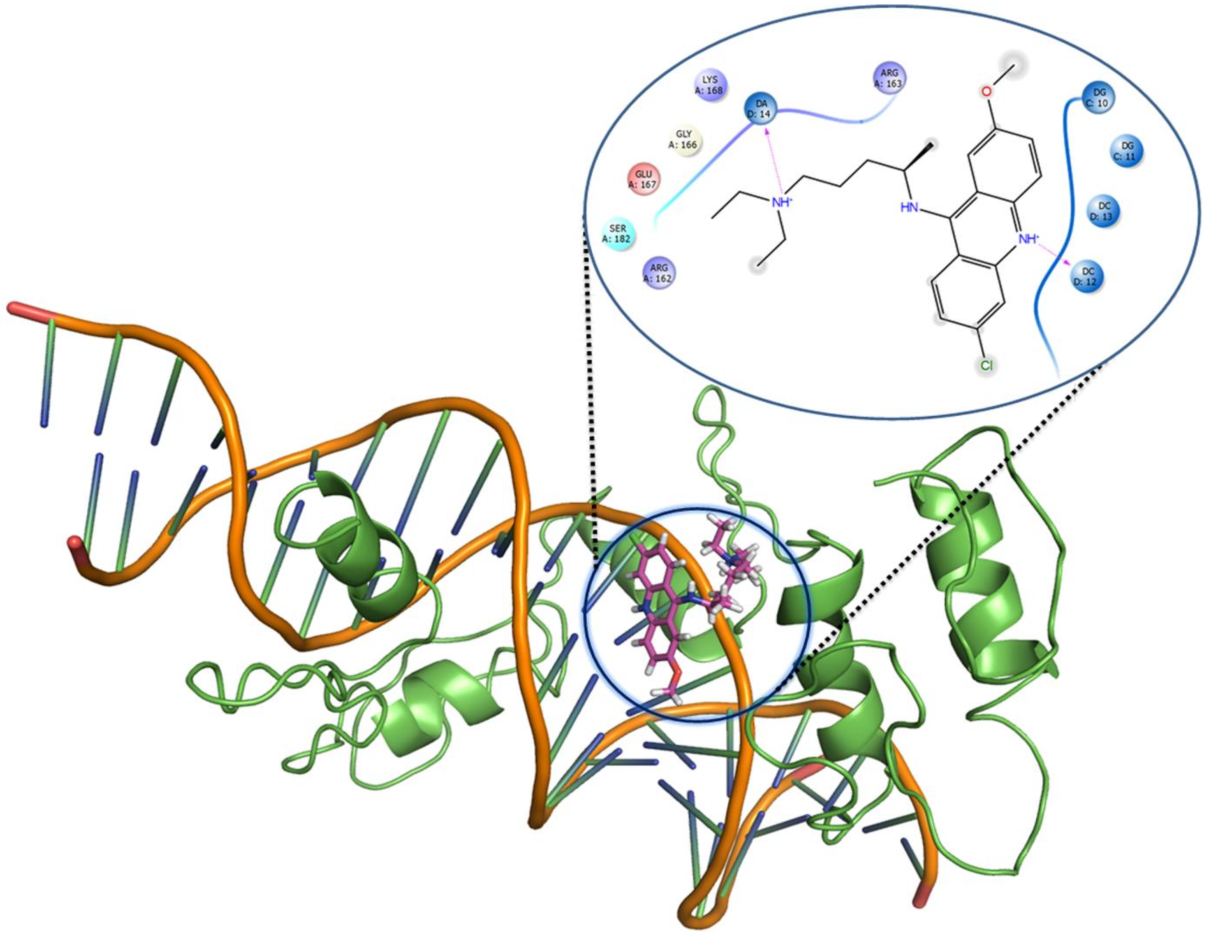

Supplementary Figure S6: Molecular docking of QC in the DNA-GLI complex. The 2D interaction diagram of QC with DNA-GLI complex shows the major interactions with DNA.

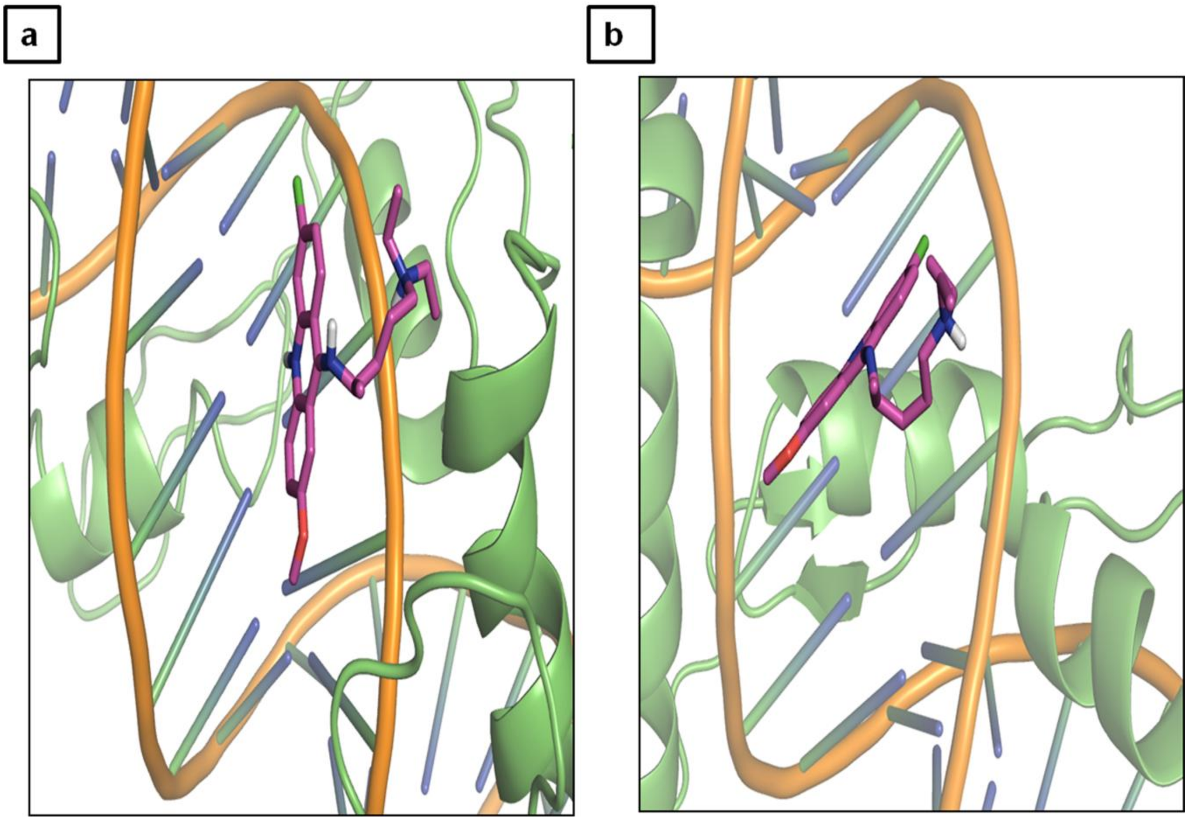

Supplementary Figure S7: DNA-GLI-QC ternary complex produced from (a) molecular docking studies and (b) after manual intercalation.

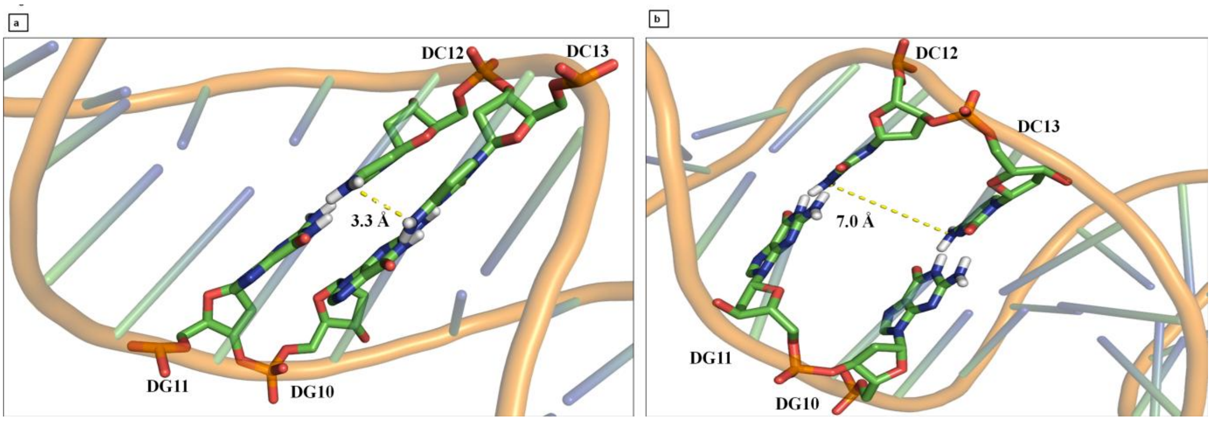

Supplementary Figure S8: Inter base pair distance in (a) DNA-GLI binary complex and (b) DNA-GLI-QC ternary complex.

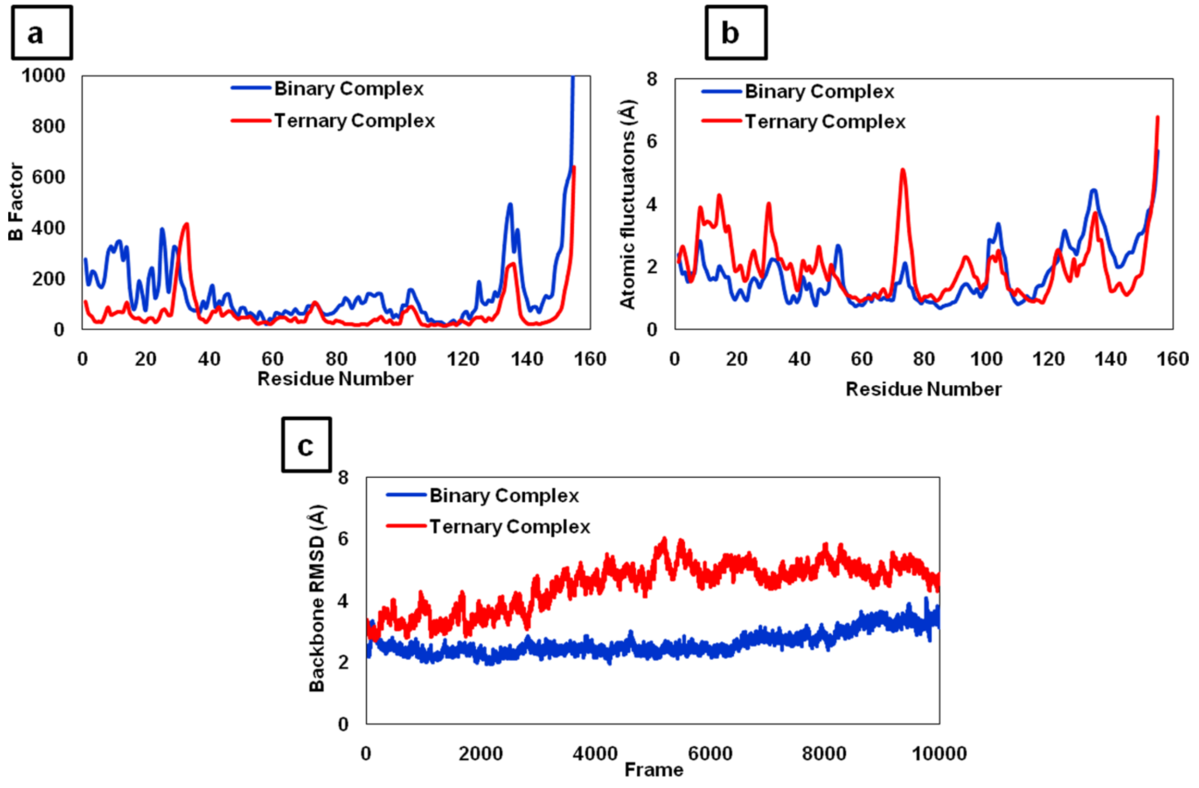

Supplementary Figure S9: The (a) backbone RMSD, (b) atomic fluctuations and (c) the B-factor for the binary (blue) and the ternary (red) complexes.

Table S1: Average binding free energy results (last 4 ns) for 2GLI (DNA+GL1) and 2GLI-QC complex (DNA-GL1-QC) along with their different energy components (**GBSA**)

System	VDW	EEL	EGB	ESURF	ΔG_{gas}	ΔG_{solv}	ΔG_{bind}
**2GLI**	-129.53	-12862.19	12799.77	-26.67	-12991.72	12773.10	-218.62 ±13.53
**2GLI+QNC**	-129.86	-11526.60	11556.27	-23.90	-11656.46	11532.37	-124.09 ± 13.85

[a]The meaning of the different terms used in this table is as follows: **VDW** = van der Waals energy as calculated by the MM force field. **EEL** = electrostatic energy as calculated by the MM force field.

EGB = the electrostatic contribution to the solvation free energy calculated by GB. **ESURF** = nonpolar solvation free energy calculated by GB. **ΔG_{gas} =** total gas phase energy i.e. sum of van der Waals and electrostatic energy from MM. **ΔG_{solv} =** total solvation free energy i.e. sum of electrostatic and nonpolar contributions from solvation. **ΔG_{bind}** = final estimated binding free energy calculated from the terms above. All the values reported above are in kcal/mol. QC-Quinacrine.

Table S2: The secondary structure content analysis of the binary (DNA-GLI) and ternary (DNA-GLI-QC) complexes

Secondary structure	Binary complex	Ternary complex
**Helix**	26.73 %	27.23 %
**Beta Sheets**	11.39 %	8.91 %
**Beta Turns**	14.36 %	12.87 %
**Random Coils**	44.55 %	48.02 %

Electro-statistical Analysis

The comparison of binary and ternary complexes revealed interesting facts about the interaction surfaces in two complexes. Some of the hydrogen bonds were weakened or broken in the ternary complex (as compared to the binary complex) e.g. hydrogen bonds with Arg146, Lys152, Arg154, Arg163, Lys179, Ser180, Arg183, Ser193, Lys198, Ser215, Lys229, Tyr231, Lys240, Thr243, Asp244 and Ser247. At the same time some new hydrogen bonds appeared in the ternary complex e.g. hydrogen bonds with Arg134, Tyr155, Arg162, Lys168, Asn186, Ser212, Gln221, Asn222, Arg223, Lys234 and His256. To understand the reason for reduced electrostatic contribution (Table S1) to complex stabilization in ternary complex, the surface potential was visualized. The results were astonishing and unveiled a very important effect of QC intercalation on the DNA-GLI complex formation. Supplementary Fig. S10a, binary complex of DNA-GLI after molecular dynamics simulation of 20 ns. Supplementary Fig. S10b and ternary complex of DNA-GLI-QC after molecular dynamics simulation of 20 ns. The results indicate that the surface potential for crystal structure and the binary complex after dynamics simulation were similar. At the DNA-GLI interface, GLI have electropositive surface potential which is complementary to the electronegative DNA sugar-phosphate backbone. Therefore the DNA-GLI complexation is facilitated. In the ternary complex (Supplementary Fig. S10c), the DNA is distorted due to QC intercalation. This further has led to the

GLI movements bringing electronegative surface close to the DNA backbone and thus producing the destabilizing effects on the ternary complex.

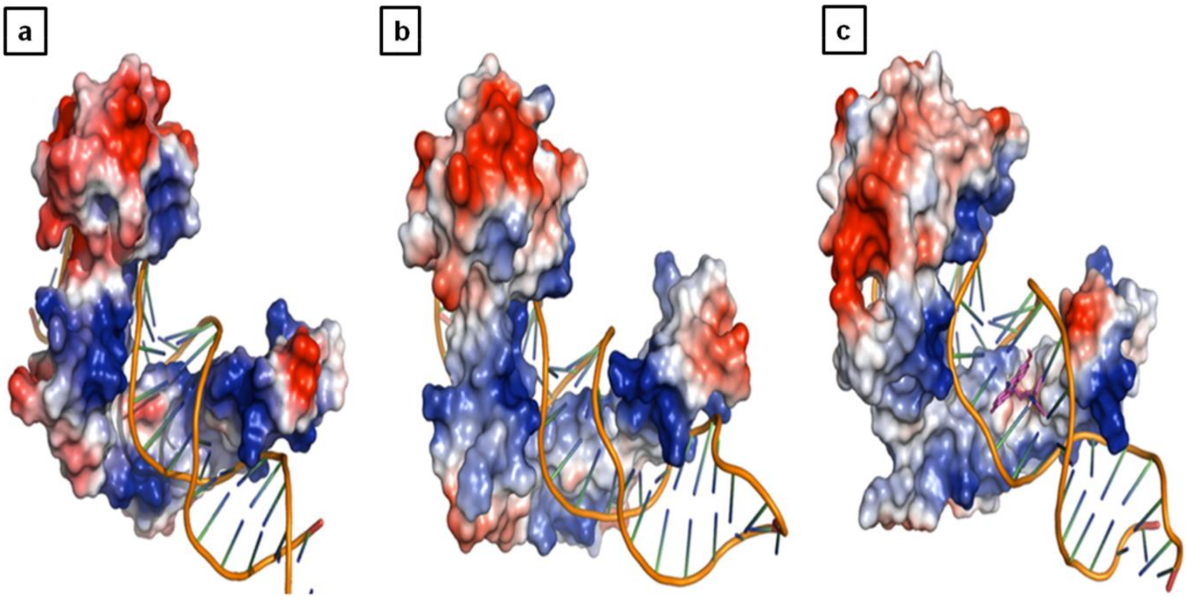

Supplementary Figure S10: Surface electro-statistics for (**a**) crystal structure of GLI-DNA complex (PDB ID 2GLI); (**b**) Binary complex of GLI-DNA complex after 20 ns molecular dynamics simulation and (**c**) Ternary complex of QC-GLI-DNA complex after 20 ns Molecular dynamics simulation.

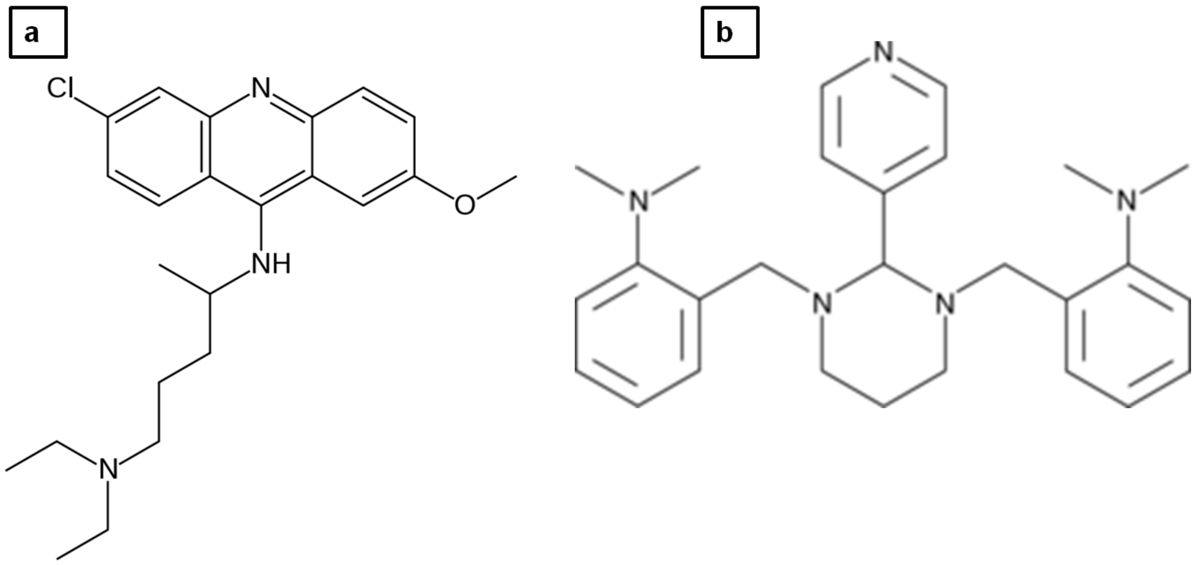

Supplementary Figure S11: 2D structure of (a) Quinacrine and (b) GANT 61

References:

1. Preet, R. *et al*. Synthesis and biological evaluation of andrographolide analogues as anti-cancer agents. *Eur. J. Med. Chem.* 85, 95-106 (2014).
2. Das, D. *et al*. 5-Fluorouracil mediated anti-cancer activity in colon cancer cells is through the induction of Adenomatous Polyposis Coli: Implication of the long-patch base excision repair pathway. *DNA Repair (Amst.)* 24, 15-25(2014).
3. Pavletich, N. P. & Pabo, C. O. Crystal structure of a five-finger GLI-DNA complex: New perspectives on zinc fingers. *Science* 261, 1701-1707 (1993).
4. *Maestro*, version 9.3; Schrödinger, LLC: New York, (2012).
5. Agyeman, A.,Jha, B. K., Mazumdar, T. & Houghton, J. A. Mode and specificity of binding of the small molecule GANT61 to GLI determines inhibition of GLI-DNA binding. *Oncotarget* 5, 4492-4503(2014).
6. Friesner, R. A. *et al*. Glide: A new approach for rapid, accurate docking and scoring. 1. Method and assessment of docking accuracy. *J. Med. Chem.* 47, 1739-1749 (2004).
7. Hossain, M. & Kumar, G. S. DNA intercalation of methylene blue and quinacrine: New insights into base and sequence specificity from structural and thermodynamic studies with polynucleotides. *Mol. BioSyst.* 5, 1311-1322(2009).
8. Ikram, M. S.*et al*. GLI2 is expressed in normal human epidermis and BCC and induces GLI1 expression by binding to its promoter. *J. Investig. Dermatol.* 122, 1503-1509 (2004).
9. *Jaguar*, version 7.5; Schrödinger, LLC: New York, (2012).
10. Bochevarov, A. D. *et al*. Jaguar: A high-performance quantum chemistry software program with strengths in life and materials sciences. *Int. J. Quantum Chem.* 113, 2110-2142(2013).
11. Dundas, J.*et al*. CASTp: Computed atlas of surface topography of proteins with structural and topographical mapping of functionally annotated residues. *Nucleic Acids Res.* 34, W116-W118(2006).
